# Modulation of cognition and neuronal plasticity in gain- and loss-of-function mouse models of the schizophrenia risk gene Tcf4

**DOI:** 10.1038/s41398-020-01026-7

**Published:** 2020-10-09

**Authors:** D. M. Badowska, M. M. Brzózka, N. Kannaiyan, C. Thomas, P. Dibaj, A. Chowdhury, H. Steffens, C. W. Turck, P. Falkai, A. Schmitt, S. Papiol, V. Scheuss, K. I. Willig, D. Martins-de-Souza, J. S. Rhee, D. Malzahn, M. J. Rossner

**Affiliations:** 1 Ludwig Maximillian’s University, Department of Psychiatry, Laboratory of Molecular Neurobiology, Munich, Germany; 2 Max Planck Institute of Experimental Medicine, Department of Neurogenetics, Göttingen, Germany; 3 Max Planck Institute of Psychiatry, Proteomics Unit, Munich, Germany; 4 Center for Nanoscale Microscopy and Molecular Physiology of the Brain, University Medical Center Göttingen, Göttingen, Germany; 5 University of Campinas, Institute of Biology, Dept of Biochemistry and Tissue Biology, Laboratory of Neuroproteomics, Campinas, Brazil; 6 Georg-August-University, University Medical Center Göttingen, Department of Genetic Epidemiology, Göttingen, Germany; 7 Present Address: mzBiostatistics, Statistical Consultancy, Göttingen, Germany

**Keywords:** Molecular neuroscience, Psychiatric disorders

## Abstract

The transcription factor *TCF4* was confirmed in several large genome-wide association studies as one of the most significant schizophrenia (SZ) susceptibility genes. Transgenic mice moderately overexpressing *Tcf4* in forebrain (*Tcf4*tg) display deficits in fear memory and sensorimotor gating. As second hit, we exposed *Tcf4*tg animals to isolation rearing (IR), chronic social defeat (SD), enriched environment (EE), or handling control (HC) conditions and examined mice with heterozygous deletion of the exon 4 (*Tcf4Ex4δ*^*+/−*^) to unravel gene-dosage effects. We applied multivariate statistics for behavioral profiling and demonstrate that IR and SD cause strong cognitive deficits of *Tcf4*tg mice, whereas EE masked the genetic vulnerability. We observed enhanced long-term depression in *Tcf4*tg mice and enhanced long-term potentiation in *Tcf4Ex4δ*^*+/−*^ mice indicating specific gene-dosage effects. *Tcf4*tg mice showed higher density of immature spines during development as assessed by STED nanoscopy and proteomic analyses of synaptosomes revealed concurrently increased levels of proteins involved in synaptic function and metabolic pathways. We conclude that environmental stress and Tcf4 misexpression precipitate cognitive deficits in 2-hit mouse models of relevance for schizophrenia.

## Introduction

*TCF4* encodes an ubiquitously expressed class I basic helix-loop-helix (bHLH) transcription factor which has been implicated in several neurodevelopmental disorders, mental retardation, intellectual disability, and schizophrenia (reviewed in ref. ^1^). The *TCF4* gene has a complex structure harboring multiple 5′ initial exons that generate longer and shorter TCF4 protein variants^2,3^. TCF4 haploinsufficiency leads to Pitt–Hopkins syndrome (PTHS) characterized by mental and developmental retardation, episodic hyperventilation, and distinct facial features^4–6^. In most cases, PTHS causing chromosomal deletions or frame shift mutations map to the 3′ end of the gene and cause TCF4 protein variants that lack the bHLH domain and/or are incapable of DNA binding^7^. A mouse mutant haploinsufficient for *Tcf4* 3′ exons 16 and 17 (which encode the bHLH domain)^8^ has been considered a model for PTHS^9^. More 5′ located deletions in *TCF4* have been identified in individuals with intellectual disability who lack other features of PTHS^2,10^.

Large genome-wide association studies (GWAS) consistently identified several non-coding single nucleotide polymorphisms (SNPs) in the 5′ located introns of the *TCF4* gene contributing to an increased risk for SZ^11–14^ and more recently also to major depressive disorder (MDD)^15,16^. *TCF4* transcript levels have been shown to be moderately increased in blood cells of SZ patients and in neurons derived from induced pluripotent stem cells (IPSCs)^17,18^ as well as in postmortem brain tissue of SZ patients^19,20^. Moreover, transgenic mice that slightly overexpress a long *Tcf4* variant (corresponding to the TCF4B variant as described by Sepp et al.^3^ in the cortex and hippocampus (*Tcf4*tg mice) display deficits in sensory gating and impairments in fear-associated learning, attentional dysfunction, and delayed adaptation in a latent inhibition task^21–23^. TCF4 is considered a constitutive interaction partner of neuronal bHLH factors and displays pleiotrophic effects^1^ and has been shown to be regulated post-trancriptionally by neuronal activity^24^. Recent shRNA-mediated knock-down analyses have provided evidence that TCF4 modulates neuronal development and function by repressing neurexins and the ion channels SCN10A and KCNQ1, respectively^25,26^. In addition, high levels of overexpression of the long TCF4 variant TCF4B by in-utero electroporation has been shown to disturb cortical laminar development in an activity-dependent fashion^27^ and haploinsufficient mice display cortical malformations as well^28^. Thus, the view emerges that loss-of TCF4 is implicated in embryonic neurodevelopment as well as postnatal neuroplasticity. Studies in humans have shown that risk alleles associated with *TCF4* may be implicated in cognitive performance and potentially also negative symptoms^18,29,30^ although sample sizes must be increased to allow definitive conclusions. According to the gene–environment interaction (GxE) model for psychiatric diseases^31,32^, it is likely that diverse environmental factors cooperate with TCF4 risk alleles and/or associated mechanisms. Thus far, only smoking has been identified as environmental risk factor modulating auditory sensory gating together with TCF4 risk alleles^33^. In patients and animal models, chronic social isolation and defeat are considered robust environmental risk factors, while intact social structures and support have been shown to ameliorate symptoms^31,34–36^. Moreover, an enriched environment positively influences rodent behavior and protects from psychopathologies^36,37^ whereas social isolation and social defeat (a model of chronic psychosocial stress) induce in mice a set of somatic, behavioral and molecular changes considered to be relevant endophenotypes of SZ^35,38–40^.

To unravel GxE interactions, we performed comprehensive behavioral phenotyping of *Tcf4*tg and wildtype mice subjected to environmental stress by post-weaning isolation rearing (IR) or social defeat (SD) in contrast to group housing in an enriched environment (EE) or handling control (HC). We also analyzed heterozygous *Tcf4* loss-of-function mice (*Tcf4*Ex4δ^+/−^) in which heterozygous deletion of exon 4 reduces the expression of long *Tcf4* transcript variants. These mice were subjected to IR to study gene-dosage effects. We complemented deep behavioral profiling by electrophysiological recordings of hippocampal plasticity, as well as structural and proteomic analyses characterizing gene-dosage dependent modulation of cognition and plasticity by TCF4.

## Materials and methods

### Behavioral profiling

The overall approach of the data calibration and dimensional reduction strategy and housing conditions were described previously^35^. Analyses were done in R software version 2.15.2 using the R-package *nlme* and R-functions *gls* and *anova*. Graphs were generated using R-package *plotrix*, exported as.eps files and edited in Adobe Illustrator CS5. Behavioral tests were in general following published procedures^22,35^. The investigator was blinded at the time of experimental procedures and the genotype decoding was performed at the end of the corresponding set of experiments. Further details and description of all mouse strains used are given in the Supplementary Information.

### Electrophysiology

Microisland autaptic culture preparation, acute brain slice preparations, and electrophysiology were performed according to published procedures^41^. LTP and LTD measurements were performed with hippocampal slices obtained from 3 to 4 week old *Tcf4*tg and *Tcf4Ex4δ*^*+/−*^ mice according to published procedures (see Supplementary Information for details).

### STED nanoscopy and proteomics

The stimulated emission depletion (STED) nanoscopy experiment was performed essentially as described^42^. Synaptosomes were isolated according to refs. ^43,44^. All Proteome analysis of cytosolic fractions (S1) and synaptosomes (S4) and the western blots were performed according to established protocols^42,45^ (see Supplemental Information for details).

## Results

### Experimental design and *Tcf4*Ex4δ^+/−^ mouse model

**Fig. 1:**
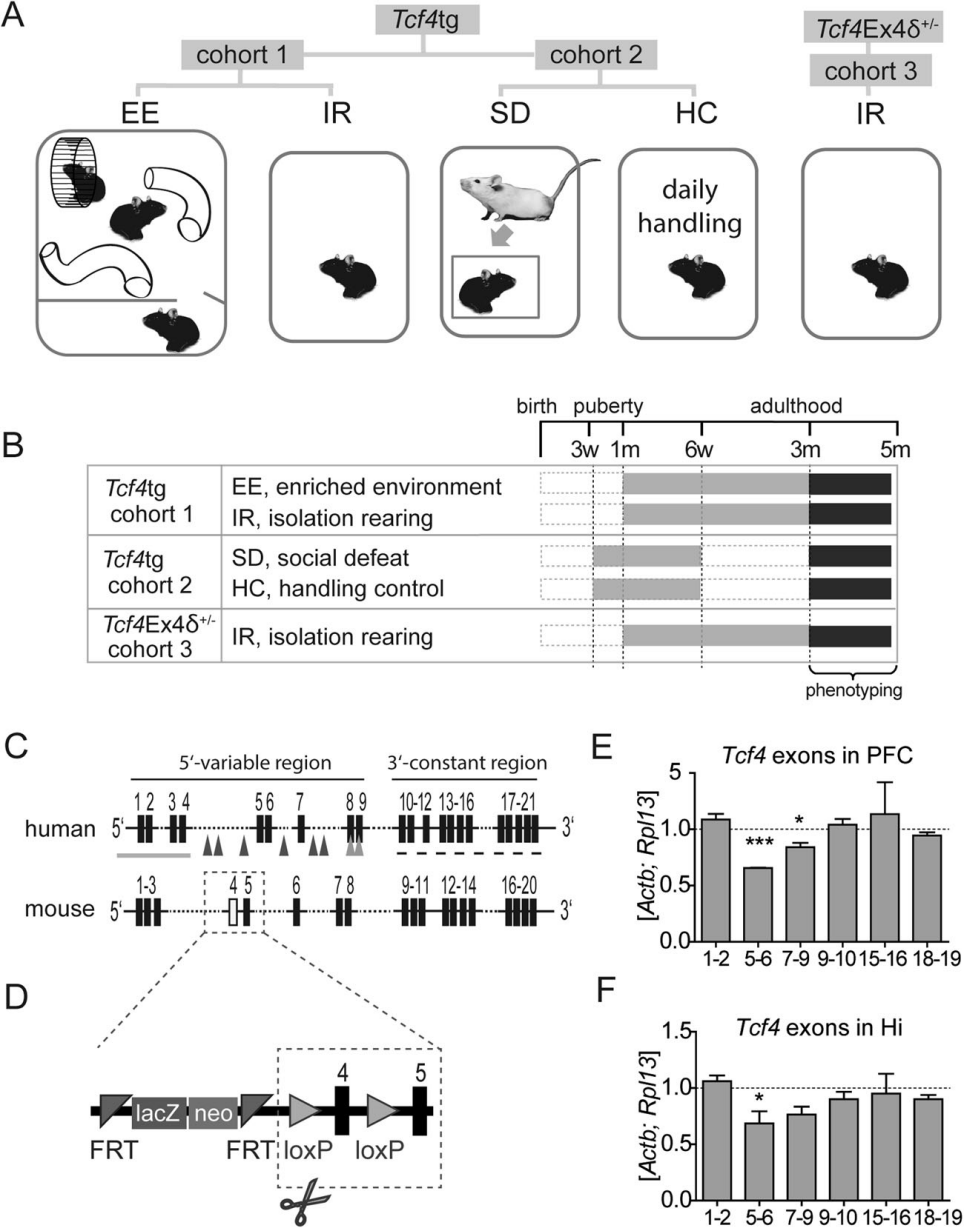
Experimental design and *Tcf4*Ex4δ^+/−^ mouse model. **a, b** Housing conditions (**a**) and experimental timeline (**b**). *Tcf4*tg and wt mice were housed in enriched environment (EE) or isolation rearing (IR) from age 4 weeks onwards (cohort 1) or were kept individually and subjected to 3 weeks of social defeat (SD) or handling control conditions (HC) from age 3 weeks onwards (cohort 2). *Tcf4*Ex4δ^+/−^ and wt animals (cohort 3) were housed in IR from age 4 weeks onwards. All mice were subjected to behavioral phenotyping from the age of 3 months. w, weeks; m, month(s). **c** Schematic representation of the human *TCF4* and mouse *Tcf4* gene. Black rectangles, exons; gray arrows, enrichment of SZ-associated risk single nucleotide polymorphisms (SNPs); gray line and gray arrow heads, 5′ region covering partial deletions associated with intellectual disability; dashed line, 3′ region with high occurrence of mutations causing mental retardation of the Pitt-Hopkins syndrome type; white rectangle, exon 4 excised in *Tcf4*Ex4δ^+/−^ mice (see main text for citations). **d** The *Tcf4*Ex4 construct containing two marker genes (*LacZ* and *neo*) flanked by FRT sites and loxP sites flanking *Tcf4* exon 4, excision of which leads to *Tcf4*Ex4δ^+/−^ genotype. loxP, lox recognition sites, FRT, flippase recognition target. **e**, **f** Relative expression levels of indicated *Tcf4* exons in prefrontal cortex (PFC; e) and hippocampus (Hi; f) of *Tcf4*Ex4δ^+/−^ mice, normalized to wt levels (dashed line) and housekeeping genes *Actb* and *Rpl13*. *Tcf4*Ex4δ^+/−^, *n* = 5; wt, *n* = 6. Data represent mean ± SEM. ***, *p* < 0.001; *, *p* < 0.05.

In this study, we combined analyses of TCF4 gain- and loss-of function mouse models: *Tcf4*tg mice, which mildly overexpress *Tcf4* in postnatal forebrain; and *Tcf4*Ex4δ^+/−^ mice, with heterozygous deletion of exon 4. To study the behavioral consequences of psychosocial stress in these mice, we analyzed three cohorts of wildtype (wt) and *Tcf4* mouse models exposed to different environmental conditions starting from the peripubertal period (Fig. 1a–d). In cohort 1, wt and *Tcf4*tg mice were exposed to enriched environment (EE) and isolation rearing (IR); in cohort 2, wt and *Tcf4*tg mice were subjected to social defeat (SD) by a 3-weeks daily exposure to an aggressive resident mouse, and to daily handling as a control (HC). In cohort 3, wt and *Tcf4*Ex4δ^+/^ were subjected to IR. Subsequently, all animals were subjected to deep behavioral phenotyping in adulthood (Fig. 1b).

By homology, exon 4 of the mouse *Tcf4* gene corresponds to exon 5 in the human *TCF4* gene which is located in the middle of the 5′ variable region (exons 1–9), which has been demonstrated to generate multiple mRNA isoforms by differential splicing and 5′ initial exons^3^ (Fig. 1c). The 3′ constant region encompasses exons 10–21 which are common to all known TCF4 splice variants^3^ (Fig. 1c). Deletions within the 5′ variable region have been shown to be associated with intellectual disability^2,10^, whereas most mutations associated with Pitt–Hopkins–Syndrome (PHS) are found in the 3′ constant region that encodes the bHLH dimerization and DNA binding domain^46^. Several SNPs thought to confer an increased risk of SZ are found in the 5′ variable region flanking human exon 5 and 6 (corresponding to murine exons 4 and 5)^12^ (Fig. 1c, d and Supplementary Fig. 1a). Therefore, we hypothesized that the *Tcf4Ex4δ*^*+/−*^ mouse line might be of relevance in the context of cognitive disability and SZ. Molecular analysis showed that truncated exon 4 of *Tcf4* was detectable only in *Tcf4Ex4δ*^*+/−*^ and not in control mice (Supplementary Fig. 1b). To measure the mRNA level of different Tcf4 exons in *Tcf4Ex4δ*^*+/−*^ mice, we performed qRT-PCR on transcripts from prefrontal cortex (PFC, Fig. 1e) and hippocampus (Hi, Fig. 1f). Expression levels of exons 1–2 in *Tcf4Ex4δ*^*+/−*^ mice were comparable to wt in both brain regions whereas exons 5–6 containing transcripts, located directly downstream of the deleted exon 4, were clearly reduced in both PFC and Hi (*p* < 0.0001 and *p* = 0.0277, t-test, respectively). The expression levels of consecutive exons were higher with increasing distance from exon 4, i.e. exons 7–9 containing transcripts showed less pronounced decrease in FCx (*p* = 0.0174, t-test) and similar tendency in Hi (Fig. 1e, f). This shows that only the long *Tcf4* transcript variants are reduced in *Tcf4*Ex4δ^+/−^ mice. In wt mice, the exons most abundant in PFC are exons 7–9, and in hippocampus exons 5–6 (Supplementary Fig. 1c, d). *Tcf4*Ex4δ^+/−^ hypomorphic mice are viable, breed well and show neither increased mortality nor major developmental impairments. We detect no abnormal facial or body features of *Tcf4*Ex4δ^+/−^ mice that might reflect PHS-like phenotype (Supplementary Fig. 1e–i).

### Tcf4 modulates cognition in a gene-dosage and environment-dependent manner

We profiled *Tcf4* gain and loss-of-function mutants in the context of G×E interactions with a comprehensive test battery assessing various behaviors’ and condensed these to behavioral “domains” and “superdomains” adapted from selected research domain criteria (RDoc) for psychiatric disease models^47^. We focused on cognitive (spatial and flexibility learning, fear memory, working memory), affective (negative and positive valence) and activity-related domains according to a multivariate statistical strategy based on stepwise data calibration and reduction combined with multiple-testing corrections (see Table 1 and Supplementary Table 1 for details) as described previously^35^. The effects discussed below always refer to multiple-testing adjusted multivariate analyses unless stated otherwise.

**Table 1:** Genetic main effects in Tcf4tg and Tcf4Ex4δ^+/−^ mice in different environments.

Superdomain	Domain	Measure	P_global_	EE environment tg/wt			IR environment tg/wt			SD environment tg/wt			IR environment *Tcf4*δEx4/wt^†^		
				Effect tg	Statistic	P	Effect tg	Statistic	P	Effect tg	Statistic	P	Effect *Tcf4*δEx4	Statistic	P
**COGNITIVE DOMAIN**		**m**	*	−0.127	*t*_80_ = −0.438	0.663	**−1.811**	***t***_**86**_ = **−2.890**	**0.005**	**−1.997**	***t***_**82**_ = **−4.372**	**<0.001**	**−1.632**	***t***_**86**_ = **−3.723**	**<0.001**
Spatial *L*earning		m	*	0.015	*t*_76_ = 0.040	0.969	−4.103	*t*_82_ = −2.397	0.019	**−3.899**	***t***_**85**_ = **−6.578**	**<0.001**	**−4.112**	***t***_**82**_ = **−6.670**	**<0.001**
	Initial learning	m	*	−0.093	*t*_50_ = −0.128	0.899	−2.598	*t*_54_ = −1.469	0.148	**−3.035**	***t***_**56**_ = **−3.378**	**0.001**	**−4.834**	***t***_**54**_ = **−4.815**	**<0.001**
	Flexibility learning	m	*	−0.621	*t*_50_ = −0.849	0.400	**−7.877**	**t**_**54**_ = **−2.919**	**0.005**	**−4.698**	***t***_**56**_ = **−6.020**	**<0.001**	**−6.862**	***t***_**54**_ = **−4.632**	**<0.001**
	Recall	m	*	0.645	*t*_50_ = 1.210	0.232	0.002	*t*_53_ = 0.004	0.997	**−4.321**	***t***_**56**_ = **−6.698**	**<0.001**	−1.456	*t*_26_ = −1.682	0.105
Fear memory		m	*	−0.413	*t*_54_ = −0.939	0.352	−0.807	*t*_58_ = −2.056	0.044	0.068	*t*_48_ = 0.179	0.858	0.806	*t*_53_ = 1.433	0.158
	Context memory	m	*	−0.417	*t*_52_ = −0.830	0.410	−0.066	*t*_58_ = −0.144	0.886	−0.318	*t*_23_ = −1.129	0.271	0.763	*t*_46_ = 1.512	0.137
	Cue memory	m	*	−0.467	*t*_52_ = −0.729	0.469	**−1.548**	***t***_**58**_ = **−3.437**	**0.001**	0.453	*t*_23_ = 0.801	0.431	0.935	*t*_54_ = 1.585	0.119
	*Social fear memory*	*m*	*ns*	–	–	–	–	–	–	*1.153*	*t*_*56*_ = *2.099*	*0.040*	–	–	–
Working memory	Working memory	u	*	0.104	*t*_26_ = 0.175	0.863	0.439	*t*_28_ = 0.581	0.566	−0.218	*t*_28_ = −0.300	0.767	**−2.032**	***t***_**28**_ = **−2.666**	**0.013**
**AFFECTIVE DOMAIN**		**m**	*	**−0.922**	***t***_**110**_ = **−3.359**	**0.001**	−0.389	*t*_121_ = −0.682	0.497	−0.159	*t*_88_ = −0.537	0.593	−0.018	*t*_118_ = −0.028	0.978
Pain sensitivity	Pain sensitivity	u	*	−1.032	*t*_26_ = −0.935	0.358	−0.773	*t*_28_ = −0.348	0.730	–	–	–	0.120	*t*_28_ = 0.098	0.923
Anxiety	Anxiety	m	*	−0.492	*t*_54_ = −1.251	0.216	−0.115	*t*_58_ = −0.490	0.626	0.371	*t*_87_ = 0.641	0.523	−0.829	*t*_83_ = −1.271	0.207
Curiosity	Curiosity	m	*	**−1.994**	***t***_**54**_ = **−2.742**	**0.008**	−1.560	t_58_ = −1.238	0.221	−0.486	*t*_57_ = −0.828	0.411	–	*F*_1.0,27.2_ = 0.251	0.621
Motivation	Motivation	m	*	−0.246	*t*_54_ = −0.403	0.689	0.115	*t*_59_ = 0.086	0.932	−0.439	*t*_58_ = −2.045	0.045	−0.142	*t*_53_ = −0.287	0.775
**ACTIVITY DOMAIN**		**m**	*	−0.418	*t*_54_ = −0.824	0.413	−0.224	*t*_60_ = −0.266	0.791	0.163	*t*_58_ = 0.560	0.577	0.490	*t*_58_ = 1.070	0.289
Hyperactivity		m	*	−0.418	*t*_54_ = −0.824	0.413	−0.224	*t*_60_ = −0.266	0.791	0.163	*t*_58_ = 0.560	0.577	0.490	*t*_58_ = 1.070	0.289
	Ambulation	m	*	0.409	*t*_110_ = 0.890	0.376	−0.768	*t*_119_ = −0.919	0.360	0.776	*t*_117_ = 1.205	0.231	0.447	*t*_113_ = 0.832	0.407
	Speed	m	*	−1.238	*t*_52_ = −1.553	0.127	−0.087	*t*_55_ = −0.071	0.944	−0.413	*t*_56_ = −1.180	0.243	0.380	*t*_56_ = 0.944	0.349

Significant differences are marked in bold and with stars in the case of Pglobal. Numbers of degrees of freedom are marked for each statistic in subscript. Statistics depicted are from the multivariate analyses (see Supplementary Table 1 for all details).

A global test revealed highly significant effects of *Tcf4* expression and environmental stress factors IR and SD, with *p* < 0.001 for all symptom classes (Supplementary Table 1). Most behavioral subdomains were significantly affected by environment, although marked differences were observed for cohort 1 (IR vs EE) and cohort 2 (HC vs SD) (see details under “Environmental Effects” column in Supplementary Table 1 and for selected tests Supplementary Fig. 2). We examined genetic main effects of *Tcf4*tg in EE, IR, SD, HC, and genetic main effects of *Tcf4*Ex4δ^+/−^ in IR, in comparison to respective wt controls. To comprehensively analyze these effects towards clinical symptom groups, we collapsed all individual behavioral tests (Supplementary Table 1) into behavioral domains that are depicted in Table 1 (for brevity, genetic main effects in HC (see Supplementary Table 1) are not displayed in Table 1 as they did not reach significance at the symptom class level). In EE, we obtained a significant difference between *Tcf4*tg and wt mice in the negative symptom class (*p* = 0.001), which was exclusively driven by the curiosity tests (rearing in the open field and hole board test, indicating reduced curiosity of *Tcf4*tg mice) (Supplementary Table 1). Note, that the effects for the individual tests failed to pass the significance threshold after Bonferroni correction whereas the multivariate and sum score analyses reached significance (Table 1 and Supplementary Table 1).

In IR (*Tcf4*tg and *Tcf4Ex4δ*^*+/−*^) and SD (*Tcf4*tg), we did not obtain significant genetic main effects of *Tcf4* alterations at the level of affective and activity domains (Table 1). Nonetheless, given the recent association of *TCF4* with depression^16^, we further inspected the subdomain level and identified that *Tcf4*tg mice exhibited under HC and SD significantly reduced fighting times in the tail suspension test monitoring depressive-like behavior (*p* < 0.001, Supplementary Table 1, Supplementary Fig. 3). The effect size of these observations, however, was not sufficient to obtain a significant result in the subdomain “Motivation” (Table 1, Supplementary Table 1).

In contrast to the affective and activity domains, the genotype significantly influenced cognitive symptoms. *Tcf4*tg mice displayed more severe cognitive impairments under IR (*Tcf4*tg: p = 0.005; *Tcf4*Ex4δ^+/−^: *p* < 0.001) and SD (*Tcf4*tg: *p* < 0.001) than respective wt controls (Table 1, Supplementary Table 1). Housing mice in EE prevented cognitive deficits in *Tcf4*tg mice (*p* = 0.663) while daily handling (HC) partially ameliorated them (*p* = 0.068) (Table 1, Supplementary Table 1). In contrast to EE, HC was insufficient to prevent deficits in spatial learning (which became most pronounced under SD, Dataset Supplementary Fig. 1), possibly because of the missing social and sensory stimulation. We further inspected the genotype effects on the cognitive subdomains by visualizing the complete spectrum of cognition-related tests in radar charts (Supplementary Fig. 4).

**Fig. 2:**
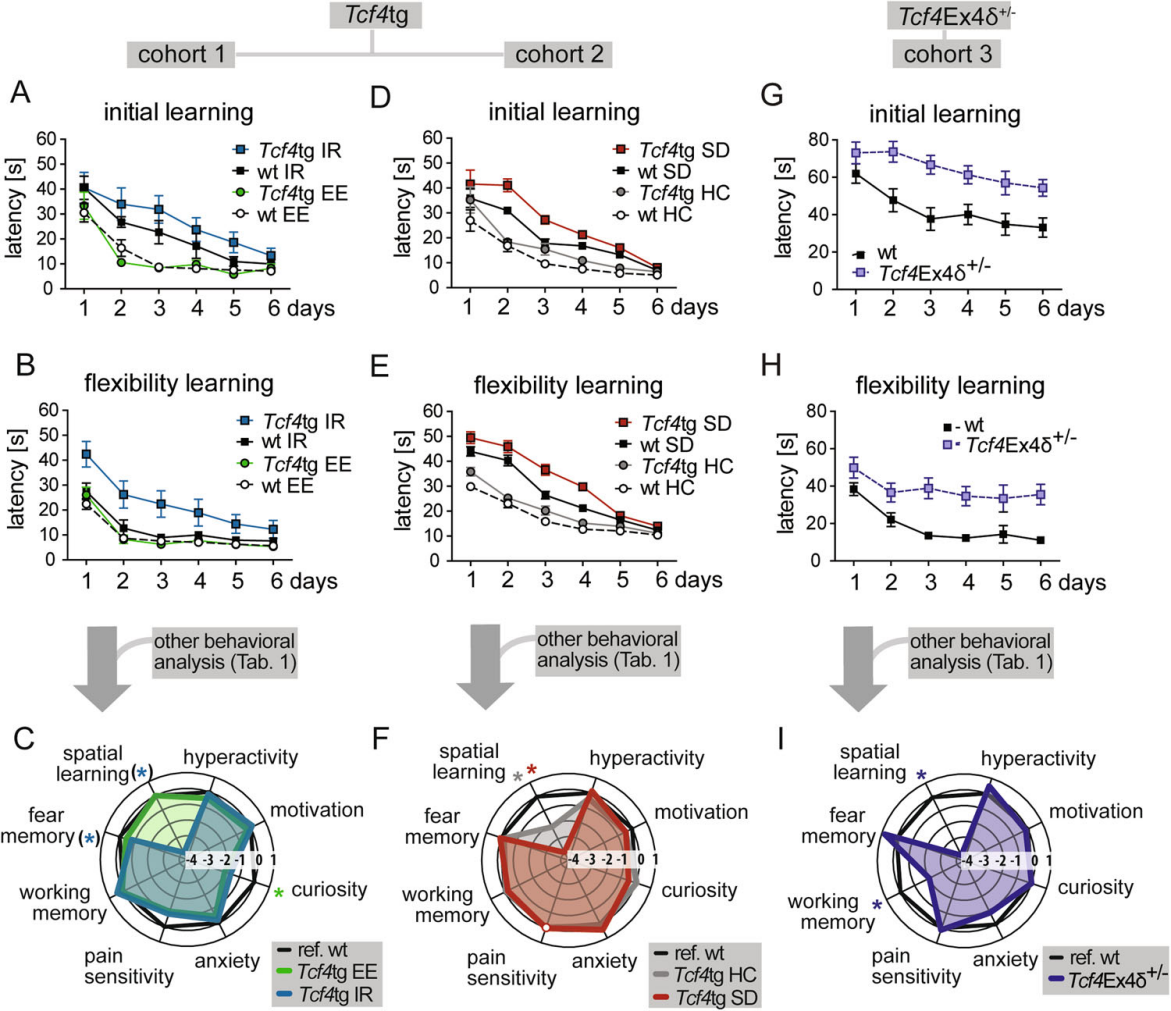
*Tcf4* gene-dosage modulates cognition in environment-dependent manner. *Tcf4*tg mice (cohort 1, **a**, **b**; cohort 2, **d**, **e**) and *Tcf4*Ex4δ^+/−^ mice (cohort 3, **g**, **h**) were submitted to spatial learning in the water maze task. Cumulated results from water maze and other behavioral profiling (Table 1) are depicted as radar charts for *Tcf4*tg (c, f) and *Tcf4*Ex4δ^+/−^ (i) mice. **a** In the initial learning task in Morris Water Maze, *Tcf4*tg and wt mice in isolation rearing (IR) learned slower than mice in enriched environment (EE) (*p* < 0.001, environmental effect), as measured by the latency to reach a hidden platform. The effect was independent of genotype (*p* = 0.210, G×E interaction test). **b** During reversal learning, *Tcf4*tg mice subjected to IR needed more time to reach the platform than IR subjected wt littermates (*p* = 0.005), indicating an impairment of cognitive flexibility. **c** Behavioral profiles show impaired spatial learning upon IR (blue) and no cognitive deficits upon EE (green) in *Tcf4*tg mice compared to wt mice from the corresponding environment (black). Green and blue stars indicate significant differences (see Table 1 and Dataset Supplementary Fig. 1 for details) between *Tcf4*tg EE (green) vs. reference (wt EE, black) and between *Tcf4*tg IR (blue) and reference (wt IR, black), respectively. IR significantly impaired cognition in *Tcf4*tg mice (cognitive symptom class: *p* = 0.005, cognitive domain flexibility learning: *p* = 0.005, cognitive trait cue memory: *p* = 0.001; all passing multiple-tesing correction). However, at the superdomain level displayed here, only nominal significance was reached (spatial learning *p* = 0.019, fear memory *p* = 0.044, blue stars in brackets). **d** During initial learning, socially defeated (SD) *Tcf4*tg mice displayed longer platform latencies than mutants from the handling control (HC) group or wt animals (*p* ≤ 0.001). *Tcf4*tg HC mice showed slightly delayed platform latencies than wt HC animals (*p* = 0.007, not reaching significance after Bonferroni correction). **e** In reversal learning (i.e. flexibility learning), *Tcf4*tg mice needed significantly more time to reach the platform than wt animals in both SD (*p* < 0.001) and HC groups (*p* = 0.002). **f** Behavioral profiles of *Tcf4*tg mice from SD and HC. Spatial learning in *Tcf4*tg mice is significantly impaired upon SD (red) and mildly in HC (gray) compared to wt mice in the corresponding conditions (black) as indicated by stars of corresponding colors (multiple-testing adjusted significance, see Table 1 and Dataset Supplementary Fig. 1 for details). Pain sensitivity was not assessed in this cohort (as indicated by white circle). **g**, **h** *Tcf4*Ex4δ^+/−^ mice housed in IR displayed higher platform latencies than wt controls in initial learning (*p* < 0.001)(**g**) and flexibility learning (*p* < 0.001)(**h**). **i** Behavioral profiles of *Tcf4*Ex4δ^+/−^ mice (blue) show that spatial learning and working memory are impaired compared to wt mice (black), as indicated by blue stars (multiple-testing adjusted significance, see Table 1 and Supplementary Table 1 for details). **a**, **b**, **d**, **e**, **g**, **h** Data represent mean ± SEM. *n* = 12–16 mice per genotype and housing conditions. See Table 1 and Dataset Supplementary Fig. 1 for detailed statistics.

The most pronounced environment-dependent cognitive deficits of the *Tcf4* mouse models manifested in spatial learning, as assessed in Morris Water Maze, in the initial learning (*Tcf4*tg in SD, *p* = 0.001, and *Tcf4*Ex4δ^+/−^ in IR, *p* < 0.001) and flexibility learning (*Tcf4*tg in IR, *p* = 0.005; *Tcf4*tg in SD, *p* < 0.001; and *Tcf4*Ex4δ^+/−^ in IR, *p* < 0.001)(Fig. 2, Supplementary Fig. 4, Table 1). The strong impact on these cognitive “superdomains” (as defined in Table 1) was visualized in radar charts for all conditions (Fig. 2c, f, i).

In summary, the behavioral profiling shows that (i) the cognitive performance is cooperatively compromised in gain and loss-of-function *Tcf4* models subjected to chronic stress and that (ii) cognitive deficits of *Tcf4*tg mice upon SD stress are not observed upon group housing in an EE.

### The impact of *Tcf4* gene dosage on neuronal plasticity

Based on the observed cognitive deficits in *Tcf4*tg and *Tcf4Ex4δ*^*+/−*^ mice, we hypothesized that basal synaptic transmission and/or neuronal plasticity may be compromised upon gain and loss of TCF4 function. To study glutamatergic neurotransmission at the single-cell level, we used the well-established autaptic culture paradigm of primary hippocampal neurons^41^ isolated from wt, *Tcf4*tg and *Tcf4Ex4δ*^*+/−*^ mice. With this system, we studied several pre- and postsynaptic properties including short-term plasticity but none of these measurements revealed a genotype-dependent alteration (Supplementary Fig. 5).

**Fig. 3:**
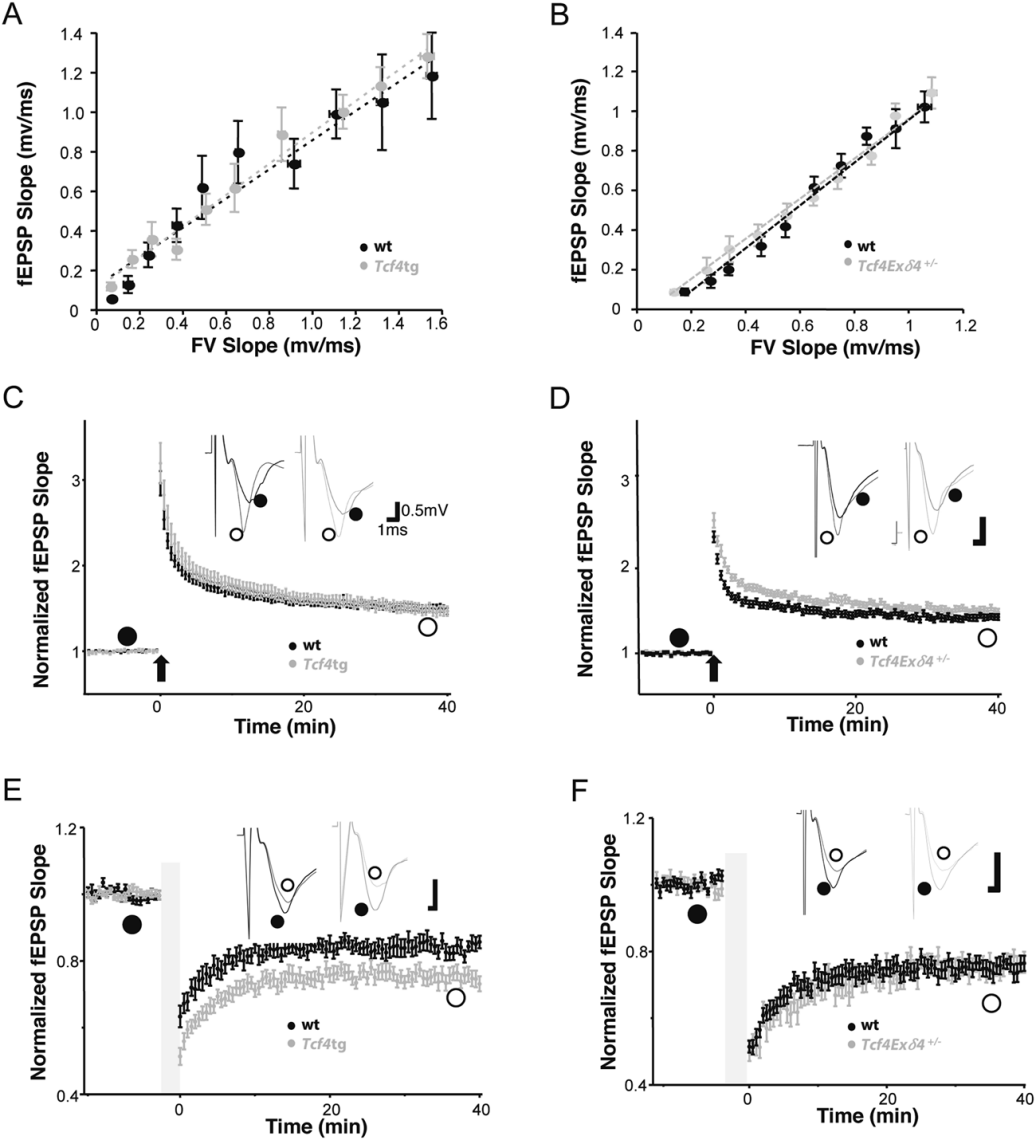
The expression level of *Tcf4* regulates long-term synaptic plasticity without change in basal synaptic transmission. Electrophysiological characterization of *Tcf4*tg (**a, c, e**) and *Tcf4*Ex4δ^+/−^ mice (b,d,f). Values from *Tcf4*tg and *Tcf4*Ex4δ^+/−^ are plotted in gray and those from littermate wt controls in black, respectively. **a, b**) Input/output relationship of excitatory synapses examined using extracellular field potential recording in the stratum radiatum layer CA1 regions of acute hippocampal slices from *Tcf4*tg and wt (**a**) and *Tcf4*Ex4δ^+/−^ and wt mice (**b**). A summary plot of the slope of the field excitatory postsynaptic potential (fEPSP) in mV/ms versus the fiber volley (FV) indicates no differences between the genotypes. *Tcf4*tg *n* = 28, wt *n* = 32; (**a**) and *Tcf4*Ex4δ^+/−^ *n* = 24, wt *n* = 15; (**b**). **c** Superimposed pooled LTP data showing the normalized changes in fEPSP slope. LTP induced by strong stimulation (100 Hz x 1) for 1 s in CA1. The fEPSP slope was measured and expressed as a mean percentage against time. The levels of LTP are unchanged in *Tcf4*tg mice (effect of genotype, *p* = 0.186). Summary graphs of LTP obtained by extracellular field recordings in CA1 stratum radiatum of acute hippocampal slices from *Tcf4*tg and wt mice (*n* = 28 and 32 slices, respectively). **d** Superimposed pooled LTP data showing the normalized changes in fEPSP slope. LTP induced by strong stimulation (100 Hz x 1) for 1 s in CA1. Summary graphs of LTP obtained by extracellular field recordings in CA1 stratum radiatum of acute hippocampal slices from *Tcf4*Ex4δ^+/−^ mice and wt controls (*n* = 22 and 34 slices, respectively) showing significantly increased LTP in *Tcf4*Ex4δ^+/−^ mice (effect of genotype, *p* < 0.001). **e** Superimposed pooled LTD data showing the normalized changes in fEPSP slope. LTD triggered by low stimulation (1 Hz) for 15 min. Summary graphs of LTD in the acute hippocampal slices from *Tcf4*tg and wt mice (*n* = 24 and 15 slices, respectively). LTD is significantly increased in *Tcf4*tg mice (effect of genotype, *p* < 0.001). **f** Superimposed pooled LTD data showing the normalized changes in fEPSP slope. LTD triggered by low stimulation (1 Hz) for 15 min. Summary graphs of LTD in the acute hippocampal slices from *Tcf4*Ex4δ^+/−^ and wt mice (*n* = 29 and 20 slices, respectively). LTD is unchanged in *Tcf4*Ex4δ^+/−^ mice (effect of genotype, *p* = 0.143). Data represent mean ± SEM. LTP, long-term potentiation; LTD, long-term depression; fEPSP, field excitatory postsynaptic potentials. wt, filled black circles; open gray circles represent *Tcf4*tg, (**c**, **e**) or *Tcf4*Ex4δ^+/−^ (**d**, **f**) vs littermate wt animals 4–5 weeks of age, respectively.

To address the functional consequences of altered *Tcf4* expression levels for synaptic transmission at the network level, we next studied synaptic transmission and plasticity at Schaffer collateral-CA1 pyramidal (SC-CA1) synapses in acute hippocampal slices from 4–5-week-old *Tcf4*tg and *Tcf4Ex4δ*^*+/−*^ mice and their wildtype littermates (Fig. 3). First, we measured input–output curves at the Schaffer collateral synapses by extracellular field recordings to reveal the impact of TCF4 on the basal synaptic transmission. There was no difference detected between slices obtained from *Tcf4*tg and *Tcf4Ex4δ*^*+/−*^ mice and corresponding wt controls (Fig. 3a, b), showing that different expression levels of TCF4 have no effect on basal synaptic transmission. Next, to understand the function of TCF4 on long-term synaptic plasticity, we measured hippocampal long-term potentiation (LTP) induced by high-frequency stimulation (100 Hz, 1 s) and long-term depression (LTD) induced by low-frequency stimulation (1 Hz, 15 min) at SC-CA1 synapses (Fig. 3c–f). LTP was unaltered in slices from *Tcf4*tg mice (mean at 35–40 min in *Tcf4*tg 150 ± 5% and in wt 151 ± 3%; *p* = 0.186) (Fig. 3c) but was significantly elevated in slices obtained from *Tcf4Ex4δ*^*+/−*^ mice (mean at 35–40 min in *Tcf4Ex4δ*^*+/−*^ 151 ± 3% versus wt 141 ± 3%; *p* < 0.001)(Fig. 3d). Moreover, *Tcf4*tg slices displayed a profound increase in LTD level (mean at 35 to 40 min in *Tcf4*tg 73 ± 2% versus wt 83 ± 2%; *p* < 0.001)(Fig. 3e), whereas in the *Tcf4*Ex4δ^+/−^ slices LTD was unchanged (mean at 35–40 min in *Tcf4*Ex4δ^+/−^ 79 ± 1% versus wt 79 ± 2%; *p* = 0.143) (Fig. 3f).

Thus, slight dysregulation of *Tcf4* expression levels does not alter basic neurotransmission but shapes long-term plasticity in hippocampal neuronal networks. Moreover, changes in *Tcf4* gene-dosage can mediate a differential impact on LTD and LTP.

### Synapse-related alterations in Tcf4tg mice

**Fig. 4:**
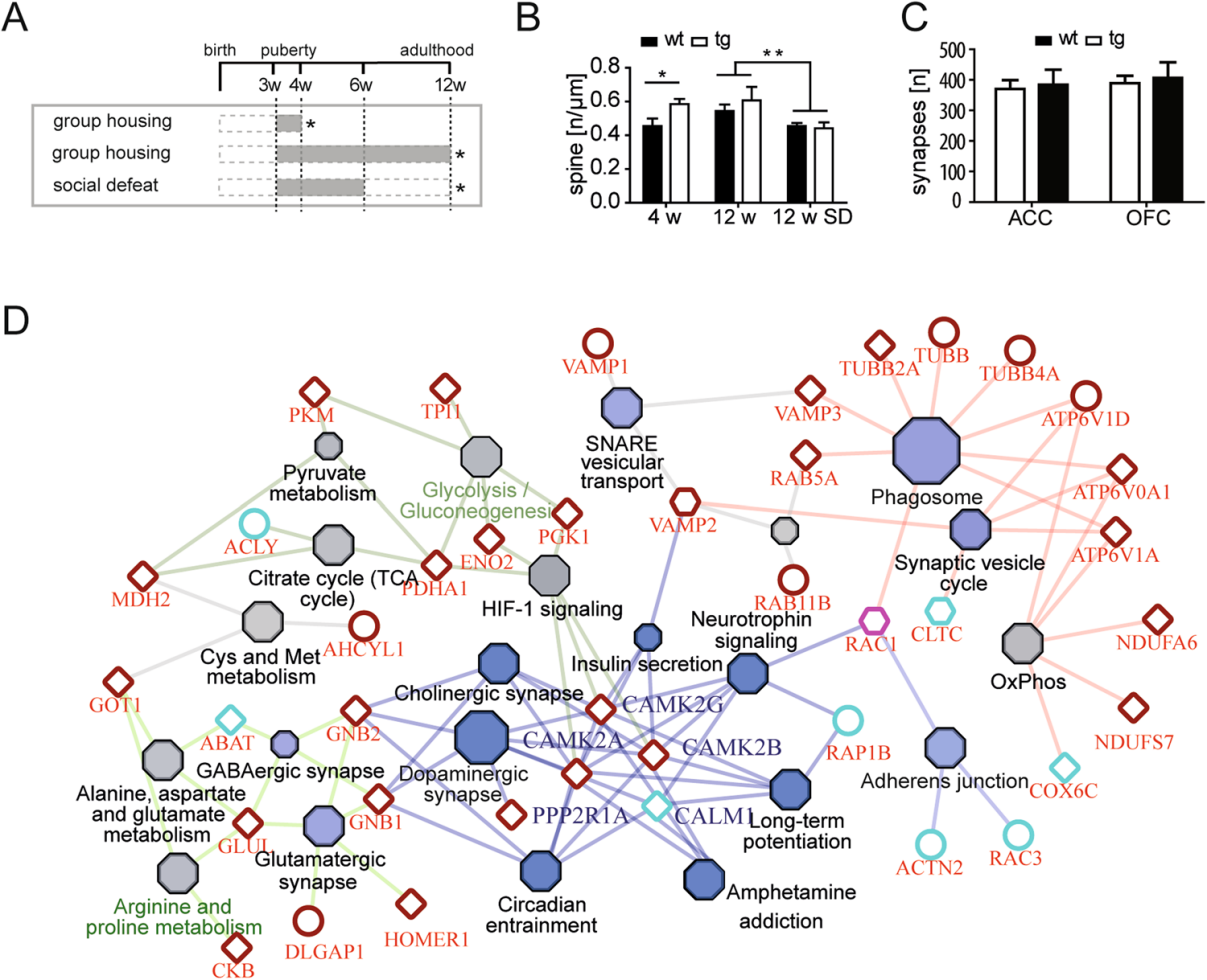
*Tcf4* overexpression leads to an increase of premature spine numbers and proteome alterations in peripubertal mice. **a** Experimental design. Group-housed *Tcf4*tg and wt mice were analyzed (*) at the age of 4 weeks (upper bar) (*n* = 4, each). Independent cohorts of *Tcf4*tg and wt mice were group-housed (middle bar) or exposed to chronic social defeat (SD, bottom bar) and analyzed at the age of 12 weeks (*). **b** Total spine density in the frontal cortex of *Tcf4*tg and wt mice was analyzed with STED nanoscopy. Spine density was significantly increased in 4-week-old *Tcf4*tg mice (*p* = 0.031). There was no difference in total spine density between the genotypes upon group housing (*p* = 0.686) or SD (*p* = 0.786) in 12-week-old mice. Subjecting mice to SD during puberty reduced total spine densities significantly (*p* = 0.003) without genotype differences. Data represent mean ± SEM. See Supplementary Fig. 6 for detailed analysis of spine types. **c** The analysis of electron-microscopic pictures for asymmetric/glutamatergic synapses reveals no differences between *Tcf4*tg and wt mice (*n* = 5, each). in the anterior cingulate cortex (ACC) nor the orbitofrontal cortex (OFC) at 4 weeks of age (*p* > 0.400). **d** Visualization of proteomic data from cytoplasmic and synaptosomal fractions isolated from frontal cortex of 4 weeks old *Tcf4*tg mice and wt controls (*n* = 4, each). Network graph of differentially regulated proteins in cytoplasmic (circular nodes), synaptosomal (diamond-shape nodes) and in both (hexagonal nodes) fractions. KEGG networks and overrepresented pathways visualized with ClueGo and CluePedia in Cytoscape are depicted as filled hexagons (blue = synapse associated pathways; grey = metabolic pathways). Primary data are from Supplementary Table 2.

Based on the altered synaptic plasticity and the evident cognitive deficits in adult *Tcf4*tg mice which were particularly prominent in the reversal “flexibility” learning paradigm (Fig. 2b, c), we aimed at identifying cellular and molecular correlates in synaptic structures. For these analyses, we focused on frontal cortical structures (anterior cingulate cortex, ACC; and medial-prefrontral/orbitofrontal cortex, OFC) implicated in associative and flexibility learning^48^ and known for its importance in schizophrenia^49^. First, we applied super-resolution STED microscopy to quantitatively assess spine morphologies and densities on dendrites from *Tcf4*tg and wt control mice where sparse numbers of neurons were genetically labeled with EYFP^42,50^. Spine morphology was analyzed in 4- and 12-week-old *Tcf4*tg and wt control mice in EE and 12-week-old *Tcf4*tg and wt mice subjected to SD (Fig. 4a). *Tcf4*tg mice showed increased total spine density at 4 weeks of age (*p* = 0.031), but no such difference was observed in 12-week-old animals (Fig. 4b). SD stress during puberty caused a significant decrease in spine density in 12-week-old mice (*p* = 0.003) independent of the genotype (Fig. 4b). We further analyzed the relative distributions and densities of five subtypes of spines with increasing morphological complexity: filopodium/stripe, stubby/stump, mushroom/racket, cup/sickle, and branched spines, as previously reported^42^ (Supplementary Fig. 6a). The relative distribution of different spine types was similar across the genotypes and experimental groups (Supplementary Fig. 6b, d, f). In accordance with the total spine analysis (Fig. 4b), spine densities of all subtypes showed a subtle yet overall significant increase in 4 weeks old *Tcf4*tg mice (*p* = 0.0055, two-way ANOVA) with the most prominent difference detected at immature “stubby/stump” like spines (*p* < 0.01, post hoc)(Supplementary Fig. 6c). Again, no significant differences between the genotypes were observed at 12-week-old animals independent of stress conditions (Supplementary Fig. 6e, g).

We performed electron microscopy on tissue sections from 4-week-old *Tcf4*tg and wt controls to assess abundance and morphologies of synapses in the ACC and OFC at the ultrastructural level (Supplementary Fig. 7). The total number of asymmetric (i.e. mature glutamatergic) synapses was not altered between the genotypes and regions (Fig. 4c). Moreover, a close inspection of several ultrastructural characteristics of synapses (shape of the postsynaptic density and active zone, number and distribution of synaptic vesicles) did not reveal any quantitative differences (Supplementary Fig. 7d–l), as was the number of mitochondria and myelinated axons unchanged (Supplementary Fig. 7m–q).

Finally, we applied a quantitative isotope-coded mass spectrometric proteomic analysis of cytoplasmic fractions and synaptosomes (enriched for pre- and postsynaptic components associated with the postsynaptic density) isolated from frontal cortex tissue of 4 weeks old *Tcf4*tg and wt littermates. After stringent filtering (fold-change >1.5/<-1.5, corr. p-val<0.05, ≥2 peptides), we identified 38 up- and 10 down-regulated proteins in the synaptosomal fraction of *Tcf4*tg mice (Supplementary Table 2). In the cytoplasmic fraction, we detected 13 up- and 24 down-regulated proteins in *Tcf4*tg (Supplementary Table 2). As expected, the overlap was low with only 4 proteins detected in both subcellular fractions (Supplementary Table 2). We performed a gene ontology pathway enrichment analysis and visualized the results as a highly connected network of pathway-node associations (Fig. 4d). This analysis identified synapse-function and interconnected metabolism associated pathways as the two major subclusters (Fig. 4d).

Taken together, our results imply that moderately dysregulated levels of TCF4 expression—50% increase in *Tcf4*tg mice^22^ and 50% decrease of the “long isoforms” in *Tcf4Ex4δ*^*+/−*^ mice—have a profound impact on higher order cognition likely caused by disturbed synaptic plasticity rather than severe structural alterations in the corresponding neuronal networks.

## Discussion

In this study, we investigated gene–environment interactions in two mouse models with slightly altered *Tcf4* transcript levels. This may mimic the changes in human *TCF4* expression levels linked to risk variants in intronic *TCF4* regions associated with SZ, MDD, and intellectual disability. By applying a “domain-oriented” deep behavioral profiling, we detected behavioral deficits in these mice, which were induced by the combination of the genetic and psychosocial risk factors. We saw that isolation rearing (IR) was more detrimental for *Tcf4*-overexpressing (*Tcf4*tg) than for wt mice, while enriched environment (EE) masked this genetic vulnerability. Similar effects were obtained when we compared *Tcf4*tg and wt mice subjected to social defeat (SD), a chronic psychosocial stress paradigm, with mice handled once daily but otherwise kept in isolation (HC). In both comparisons, most behavioral domains were affected except activity, which was assessed by “ambulation” (i.e. speed and distance running) in several behavioral tests. These were used as the only surrogate parameter of a hyperactive state often observed during a psychosis-associated phase in patients, possibly reflecting an aspect of positive symptoms^51^. This clearly indicates a limitation of rodent models because prototypic positive symptoms cannot be investigated. Nonetheless, we believe that our findings of strong GxE interaction provide evidence that the “2-hit” *Tcf4*/SD and *Tcf4*/IR approaches represent valid mouse models for the cognitive subdomain of relevance for SZ and possibly also MDD. *Tcf4*tg mice used for behavior were on F1 hybrid background while *Tcf4*Ex4δ^+/−^ were kept on a pure C57bl6 background. Thus, we cannot formally rule that this may have an impact on the direct comparison of the different lines, we consider this as rather unlikely since we have previously shown that *Tcf4*tg mice backcrossed for several generations on the C57bl6 background performed highly similar in behavioral testing compared to F1 hybrids^22^. The “pairing” of risk gene alterations with environmental stress conditions has been suggested as promising strategy to improve validity of psychiatric mouse models^52^. Given the strong associations of *TCF4* and psychosocial stress with schizophrenia and MDD, it seems likely that relevant mechanisms are affected in our 2-hit *Tcf4* gain- and loss-of-function mouse models.

*TCF4* has recently been identified by the PsychEncode consortium as a key “hub” gene in human brain development^28^, and was found to be expressed at increased levels in the Hi and PFC of SZ patients from independent postmortem sample collections^53^. On the other hand, both increased and reduced *TCF4* expression in blood has been detected in independent studies and subject groups suffering from psychosis^18,54^, and reduced levels were found in recurrent MDD^55^. However, this variability of disease-associated changes of *TCF4* expression in postmortem brain and blood samples does not allow conclusions on causal mechanisms and may be secondary or independent of the proposed neurodevelopmental alterations caused by *TCF4* misexpression. Nonetheless, it may be possible that dichotomous patterns of deregulated expression of risk genes in SZ and MDD, including *TCF4*, may occur during later neurodevelopmental stages, converging at the level of synaptic dysfunctions, which are major molecular pathways detected in the recent comprehensive GWAS analyses of SZ and MDD^11,15^. Our analysis supports this assumption, because it demonstrates altered structural and functional synaptic plasticity potentially at the level of developmental spine dynamics, enhanced hippocampal LTD (*Tcf4*tg) and LTP (*Tcf4*Ex4δ^+/−^) and changes in the molecular composition of synaptosomes in *Tcf4* gain-of-function (*Tcf4*tg) mouse model. The enhanced LTP in *Tcf4*Ex4δ^+/−^ mice is in agreement with the same phenotype observed in other *Tcf4* loss-of-function mutants^9,56^. Moreover, reduction of spine densities has recently been described in the cortex and Hi in loss-of-function mice^57^. In these animals, more immature synapses of the stubby subtype were affected in the cortex, which may indicate an inverse gene-dosage dependent phenotype when compared to the increased number of stubby-like synapses that we observed in 4 weeks old *Tcf4*tg mice (Supplementary Fig. 6c). However, we detected no changes at the ultrastructural level of pre- and post-synapses and no alterations of electrophysiological features in individual neurons when cultured in isolation. This indicates that a slight deregulation of *Tcf4* expression causes rather subtle changes in neuronal plasticity and during subsequent network refinement upon interaction with psychosocial stressors. Since heterozygous null mutant mice also display structural as well as neuroanatomical aberrations likely changing micro- and macroconnectivity, it may as well be possible that structural changes also contribute to the cognitive alterations observed in our study^28,58^. Our study thus provides the basis to further dissect the molecular and cellular mechanisms misrouted upon deregulated *Tcf4* expression, e.g. by identifying gene regulatory networks with transcriptome profiling. The switch from enhanced LTP to increased LTD in *Tcf4* gain- versus loss-of-function mouse models is to our knowledge the first example where such a gene-dosage dependent relationship has been described.

Calcium signals from ligand and voltage-gated channels play a central role in coupling synaptic activity to the downstream signaling cascades, and several members of both groups (NMDA- and AMPA-receptors and L-type Ca-channels, in particular) have been strongly associated with psychotic disorders^11,15^. The relationship between the strength of synaptic activity and the equivalent calcium signal on one hand and the sign (potentiation versus depression) and degree on the other is described by the Bienenstock-Cooper-Munro (BCM) model^59,60^. This model is in agreement with the findings that sliding calcium levels activate different calcium/calmodulin-dependent protein kinases and/or other calcium-modulated synaptic proteins, which partially determine the differential induction of LTP or LTD^61^. Several such candidates have been identified in our proteome analysis. Based on our findings, follow up experiments are needed to substantiate the hypothesis that *Tcf4* gene dosage determines postsynaptic Ca^2+^ levels and to identify the responsible target genes of TCF4. We conclude that the presented 2-hit mouse models represent valid tools for pre-clinical treatment trials, e.g. with cognitive enhancers that target cognitive processing units of relevance for affective and non-affective psychoses.

## Supplementary information

Supplemental Material

Supplemental Table 1

Supplemental Table 2
