# Supplementary material for: Modulation of cognition and neuronal plasticity in gain- and loss-of-function mouse models of the schizophrenia risk gene Tcf4
Source: Transl Psychiatry. 2020 Oct 9;10:343. doi: 10.1038/s41398-020-01026-7 (PMC7547694; doi:10.1038/s41398-020-01026-7)
Supplement: Supplementary file 1 — Supplemental Material [file 41398_2020_1026_MOESM1_ESM.docx]

**Supplemental Material to**

**Modulation of Cognition and Neuronal Plasticity in gain- and loss-of-function mouse models of the schizophrenia risk gene Tcf4**

Badowska DM^1,2^ *, Brzózka MM^1,2^ *, Kannaiyan N^1^, Thomas C^2^, Dibaj P^2^, Chowdhury A^4^, Steffens H^5^, Turck CW^4^, Falkai P^1^, Schmitt A^1^, Papiol S^1^, Scheuss V^1^, Willig KI^2,5^, Martins-de-Souza D^4,6^, Rhee JS^2^, and Malzahn D^3‡^, Rossner MJ^1,2^

^1^Ludwig Maximillian’s University, Department of Psychiatry, Laboratory of Molecular Neurobiology, Munich, Germany

^2^Max Planck Institute of Experimental Medicine, Department of Neurogenetics, Göttingen, Germany

^3^Georg-August-University, University Medical Center Göttingen, Department of Genetic Epidemiology, Göttingen, Germany

^‡^present address: mzBiostatistics, Statistical Consultancy, Göttingen, Germany

^4^Max Planck Institute of Psychiatry, Proteomics Unit, Munich, Germany

^5^Center for Nanoscale Microscopy and Molecular Physiology of the Brain, University Medical Center Göttingen, Göttingen, Germany

^6^University of Campinas, Institute of Biology, Dept of Biochemistry and Tissue Biology, Laboratory of Neuroproteomics, Campinas, Brazil

* contributed equally

corresponding author:

Molecular and Behavioral Neurobiology and Department of Psychiatry

Nussbaumstr.7, 80336 München, Germany

Phone: +49 89 4400 55891

[Moritz.Rossner@med.uni-muenchen.de](mailto:Moritz.Rossner@med.uni-muenchen.de)

Contents:

Supplemental Figures

Supplemental Material and Methods

Supplemental References

**Supplemental Figures**

**
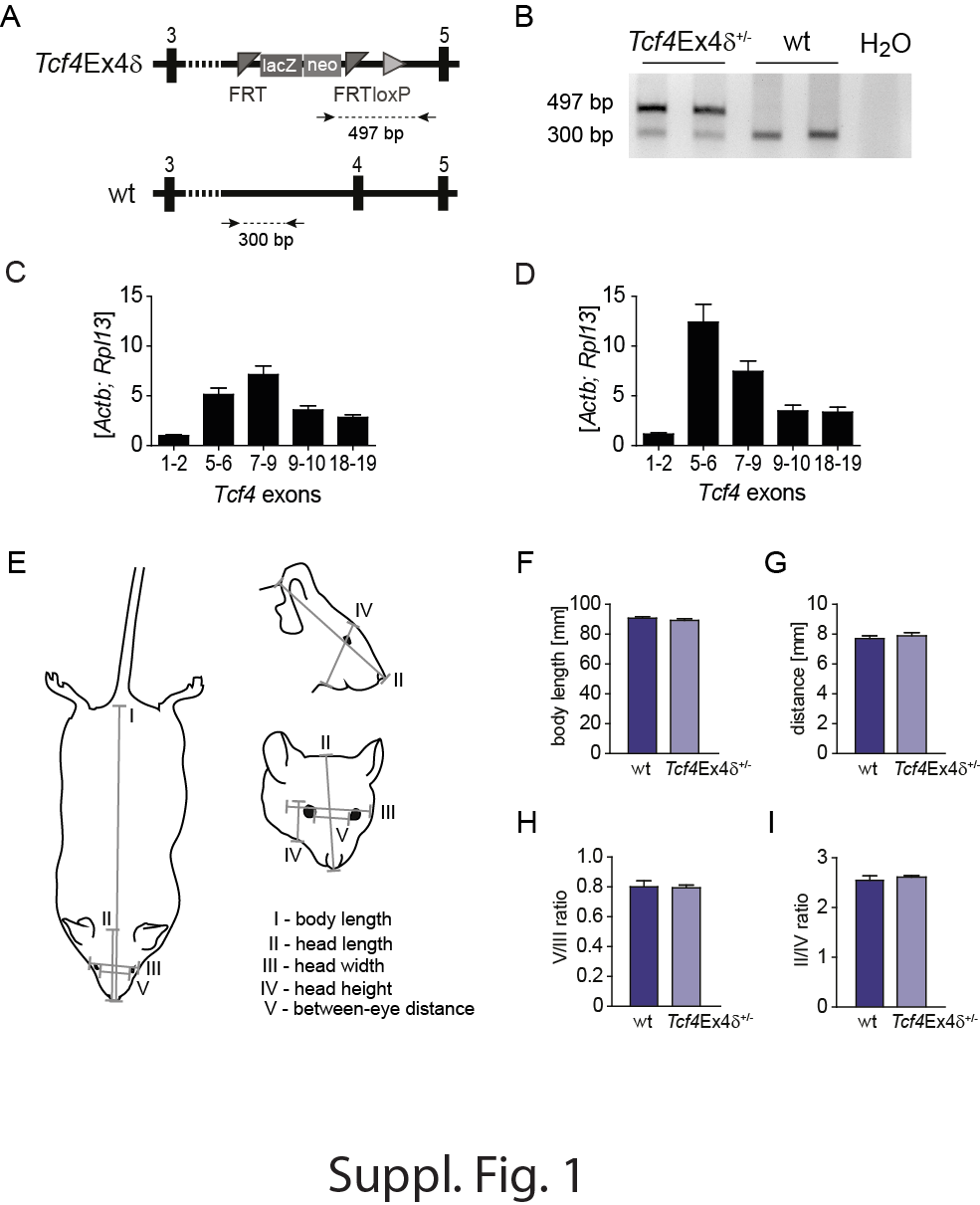
**

**Suppl. Fig. 1 Molecular and morphological characterization of *Tcf4*Ex4δ^+/-^ mice.**

a-b) Schematic drawing of regions targeted by primers (a) and gel electrophoresis yielding *Tcf4*Ex4δ related product of 497 base pairs (bp) and 300 bp wt related allele (b). In *Tcf4*Ex4δ^+/-^ mice both bands are visible, whereas only the 300 bp allele is amplified in wt animals.

c-d) Relative expression levels of indicated *Tcf4* exons in prefrontal cortex (c) and hippocampus (d) of wt mice. Data are normalized to housekeeping genes *Actb* and *Rpl13*, expressed relative to exons 1-2 and corrected for differences in PCR efficiency. In the Hi, the highest expression was observed for *Tcf4* exons 5-6, which are expressed in PFC at lower levels. Data represent mean±SEM. n=6 biological and 3 technical replicates.

e-i) *Tcf4*Ex4δ ^+/-^ mouse morphometrics. e) Measured dimensions. f-i) *Tcf4*Ex4δ^+-^ mice display no prominent morphological alterations in body length (f), the distance between the eyes (g), the between-eyes distance to head width ratio (h) and head length to head high ratio (i). Data represent mean±SEM. *Tcf4*Ex4δ^+/-^, n=10; wt, n=7 (p>0.200). In contrast, cranio-facial abnormalities and prenatal mortality have been observed in corresponding homozygous exon 4 deletion mice reminiscent of PTHS-like phenotypes, which are absent in heterozygous hypomorphic mice (www.mousephenotype.org/data/genes/MGI:98506#section-associations).


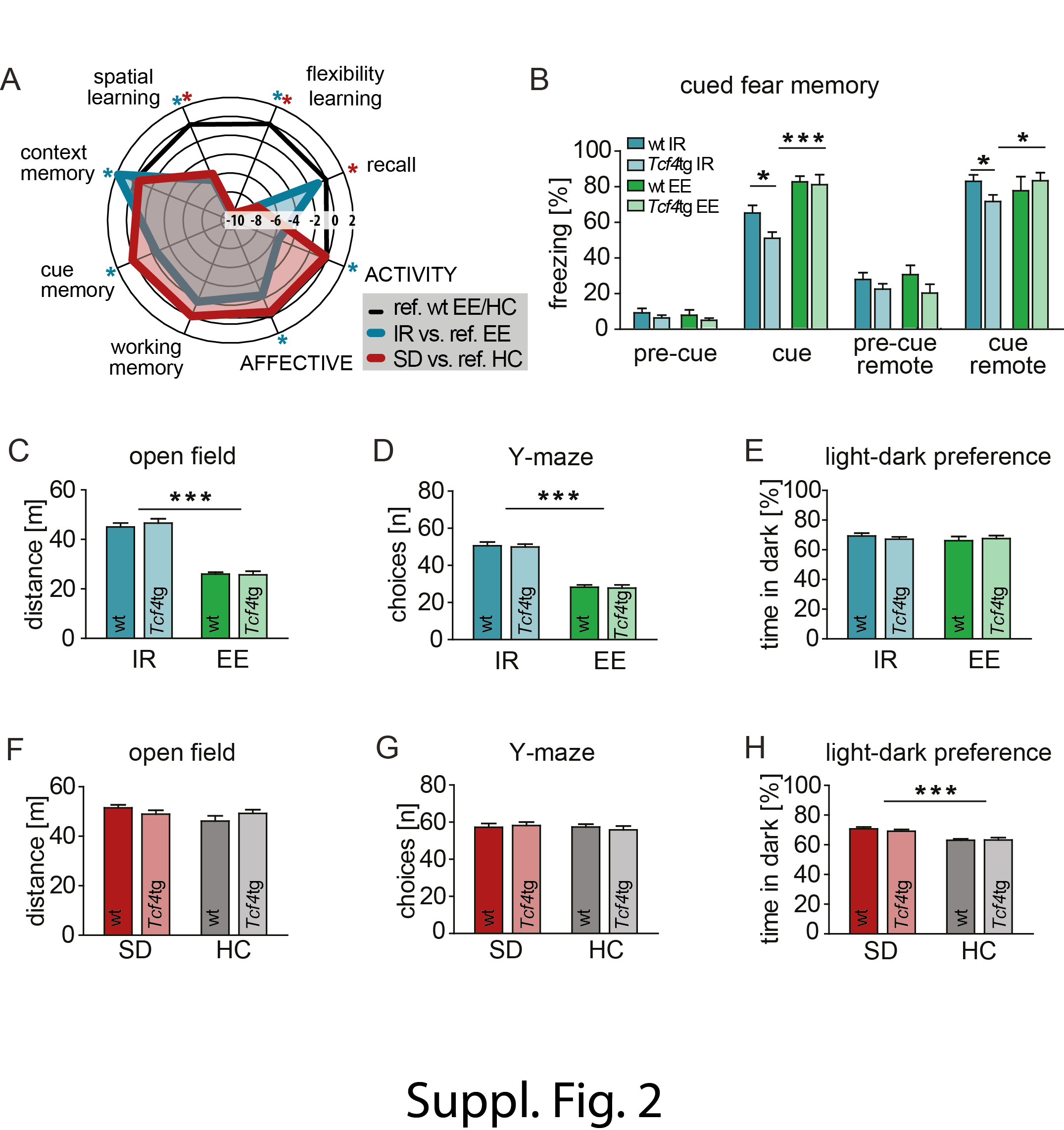


**Suppl. Fig. 2 Isolation rearing and social defeat strongly influence murine behavior.**

a) Radar charts showing environmental effects of isolation rearing (IR, blue) and social defeat (SD, red) on cognition (domain level) and positive and negative symptoms (symptom class level), relative to corresponding reference environments (black): enriched environment (EE) for IR and handling control (HC) for SD. Environmental main effects were estimated for cohort 1 and 2, in a 2-factorial design with genotype as second factor. Significant environmental effects on murine behavior are indicated by stars in corresponding colors (see Table 1 and Dataset Suppl. Fig. 1 for details on statistics).

b) IR significantly reduced freezing behavior 48 hours after acquisition in fear conditioning test (p<0.001, environmental effect: blue vs. green groups). Isolated *Tcf4*tg animals showed more prominent impairment than isolated wt animals 48 h (p<0.05) and 1 month after conditioning (p<0.05; light blue vs. dark blue). EE prevented memory deficits in *Tcf4*tg mice (green bars).

c-d) Isolation rearing significantly increased locomotor activity measured by the distance covered in the open field (p<0.001) and the number of arms explored in Y-maze (p<0.001) regardless of genotype.

e) IR did not influence time spent in the dark compartment during the light-dark preference test (p> 0.500).

f-g) SD had no significant effect on the distance covered in the open field and arm exploration in Y-maze (p> 0.400).

h) SD significantly increased time which mice spent in the dark compartment in light-dark preference test independent of genotype (p=0.003).

b-h) Data represent mean±SEM. n=12-16 mice per genotype. * indicates p<0.05, *** indicates p≤0.003; see Table 1 and Dataset Suppl. Fig. 1 for all values and additional details on statistics.


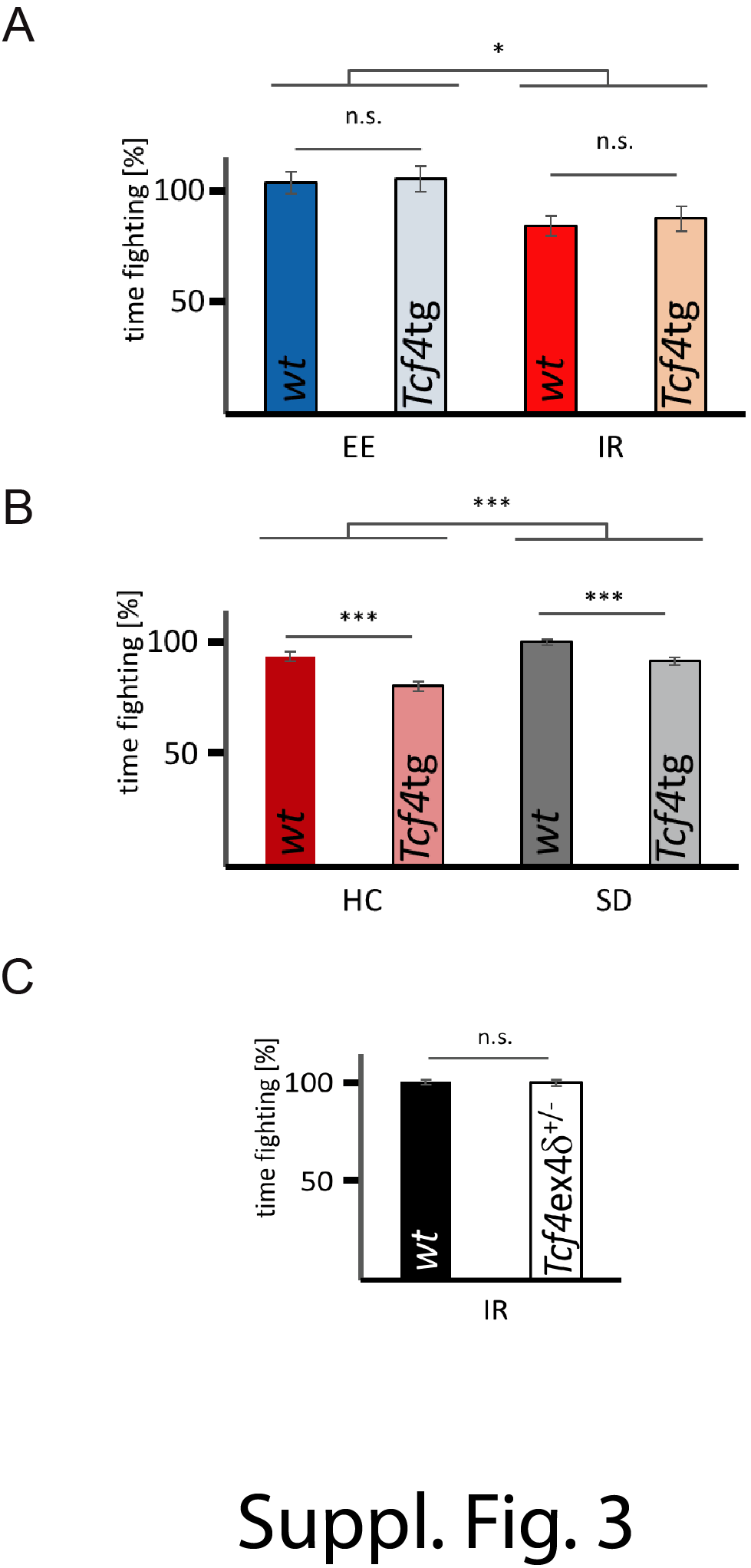


**Suppl. Fig. 3 Depressive-like behavior of Tcf4tg mice in the tail suspension test.**

a-c) The tail suspension test was used to measure depressive-like behavior in cohorts 1 (a), -2 (b) and -3 (c) comparing enriched environment (EE) and isolated rearing (IR) (a) and handling control (HC) as well as social defeat (SD) (b) in wt and *Tcf4*tg mice and wt, and *Tcf4*Ex4δ^+/-^ mice under IR conditions (c).

a) Mice housed under IR compared to EE showed reduced levels of time fighting in the tail suspension test (p<0.05), indicating more depressive-like behavior. No significant genotype-dependent changes were observed.

b) Mice housed under SD compared to HC showed significantly reduced levels of time fighting in the tail suspension test (p<0.001), and *Tcf4*tg mice under both conditions displayed shorter fighting times than wt (p<0.001).

c) No significant changes were obtained when comparing wt and *Tcf4*Ex4δ^+/-^ in the tail suspension test.

Data represent mean±SEM. n=12-16 mice per genotype. * indicates p<0.05, *** indicates p<0.001; see Table 1 and Dataset Suppl. Fig. 1 for all values and additional details on statistics.


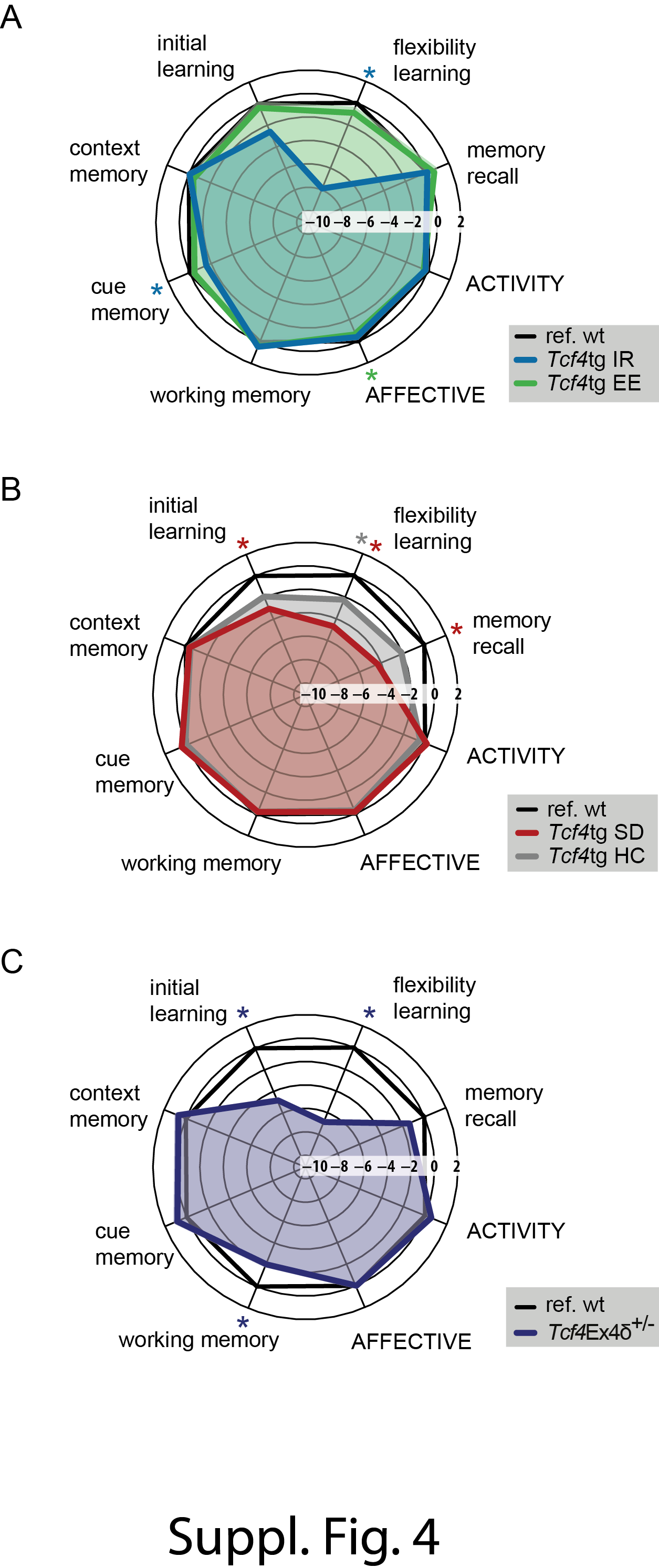


**Suppl. Fig. 4 Profile of cognitive deficits in *Tcf4*tg and *Tcf4*Ex4δ^+/-^ mice**

Radar charts showing a detailed view on cognitive symptoms (domain level) and overall effects in positive and negative symptom classes. Colored lines represent effect sizes in *Tcf4*tg or *Tcf4*Ex4δ^+/-^ mice compared to littermate wt mice from the corresponding environment (black).

a) Behavioral profiles of cohort 1: *Tcf4*tg in isolation rearing (IR; blue) or enriched environment (EE; green), and wt animals from corresponding housing conditions (black). Upon IR, *Tcf4*tg mice showed pronounced impairment of flexibility learning and cue fear memory (blue stars: multiple-testing adjusted significant). EE prevented cognitive deficits.

b) Behavioral profiles of cohort 2: *Tcf4*tg mice subjected to social defeat (SD, red) or handling controls (HC, gray) compared with wt mice from corresponding conditions (black). Upon SD, *Tcf4*tg mice display strong impairments of initial spatial and flexibility learning, and spatial memory recall (red stars: multiple-testing adjusted significant), compared to wt. HC-induced deficits of *Tcf4*tg mice were milder, reaching multiple-testing adjusted significance only for the reversal learning task (flexibility learning).

c) Behavioral profiles of cohort 3: *Tcf4*Ex4δ^+/-^ (violet) and wt (black) mice in IR. *Tcf4*Ex4δ^+/-^ animals showed impairments of spatial initial and flexibility learning, and deficits of working memory (violet stars: multiple-testing adjusted significant).

n= 12-16 mice per genotype and housing conditions. * indicates multiple-testing adjusted significance, see Table 1 and Dataset Suppl. Fig. 1 for all values and additional details on statistics.

**
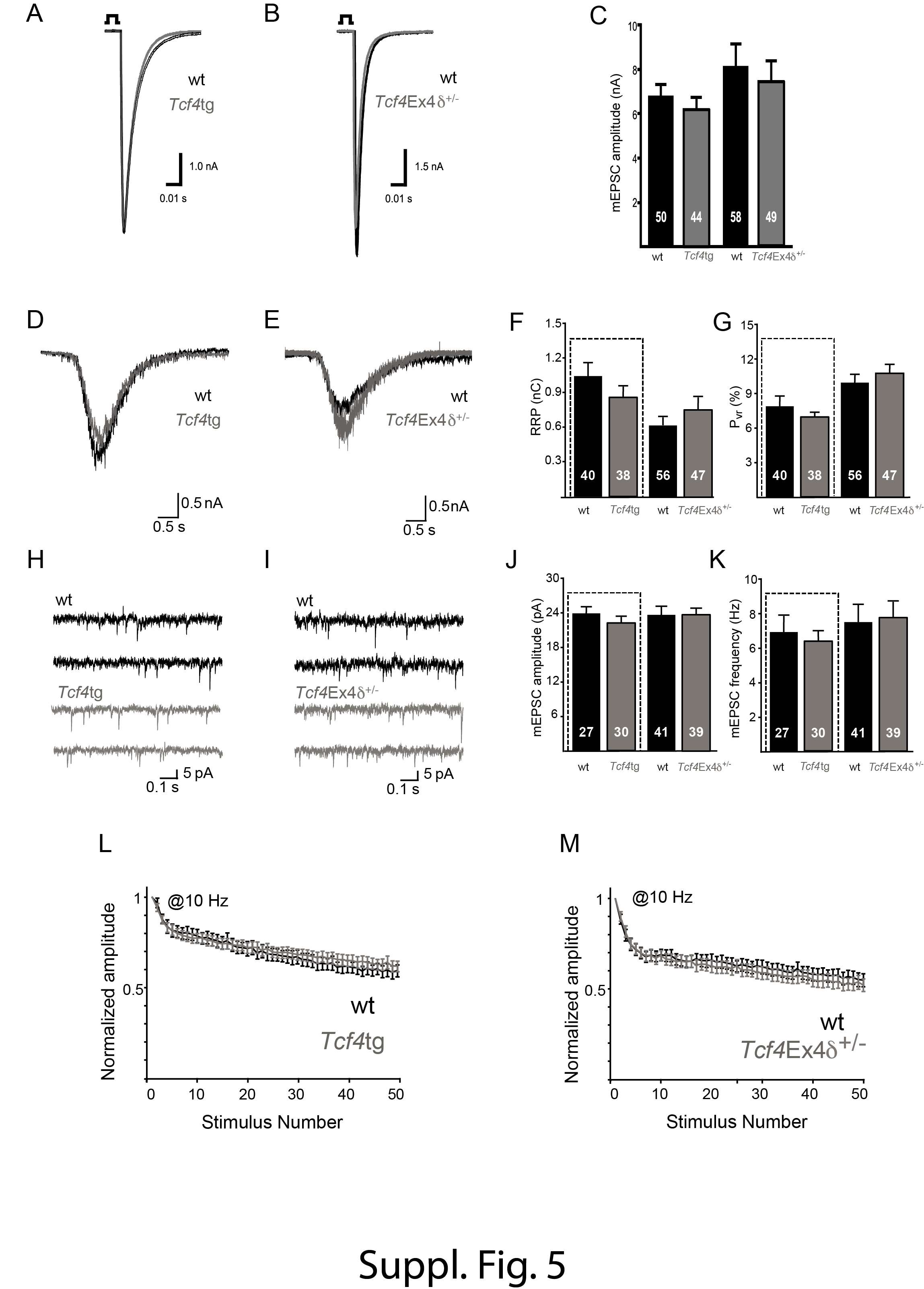
**

**Suppl. Fig. 5 Glutamatergic synaptic transmission in autaptic neurons is not affected by *Tcf4* gene dosage**

a-c) Measurement of the overall synaptic function depicted as EPSC amplitudes evoked by a 2-milisecond depolarizing pulse. Samples traces are depicted for *Tcf4*tg in a) and *Tcf4Ex4δ^+/-^* in b). c) No significant alterations were found neither in *Tcf4*tg (*Tcf4* WT= -6.782 nA ± 0.5048 n=50, *Tcf4* tg= -6.230 nA ± 0.5408 n= 44, p=457) nor in *Tcf4Ex4δ^+/-^* neurons (*Tcf4* WT= -8.161 nA ± 1.016 n= 58, *Tcf4Ex4δ^+/-^* = -7.406 nA ± 0.9288 n=49, p=0.986).

d-f) Measurement of the pool of readily-releasable vesicles (RRP) by application of 500 mM sucrose. Sample traces are shown for wt and *Tcf4*tg (d), and wt and *Tcf4*Ex4δ^+/-^ (e), no difference was observed in the transferred charge in any of the mutations (wt, 1.054 nC ± 0.1186 n=40, *Tcf4*tg, 0.866 nC ± 0.1012 n=38, p=0.288; wt, 0.6165 nC ± 0.076 n=56; *Tcf4*Ex4δ^+/-^, 0.7516 nC ± 0.1151 n=47, p=0.612) (f).

g) Probability of vesicle release calculated as the ratio between the RRP charge and the EPSC charge showed no changes across genotypes. (wt, 7.881% ± 0.8981 n=40, *Tcf4*tg, 6.958% ± 0.4271 n=38, p=0.844*;* wt, 9.962% ± 0.7162 n=56, *Tcf4*Ex4δ^+/-^, 10.79% ± 0.7667 n=47, p=0.453).

h-k) Quantification of the spontaneous glutamate release in the presence of the tetrodotoxin (TTX). Sample traces are shown for wt and *Tcf4*tg (in h), and wt and *Tcf4*Ex4δ^+/-^ (in i), with no evidence of change observed in the amplitude or the frequency of miniature EPSCs by genotype (j) (Amplitude: wt, 23.98 pA± 1.235 n= 27; *Tcf4*tg= 22.15 pA ± 1.095 n=30, p=0.271; wt, 23.94 pA ± 1.480 n=41; *Tcf4*Ex4δ^+/-^ = 23.95 pA ± 1.193 n=39, p=0.626. (k) Frequency: wt, 6.974 Hz ± 09363 n=27, *Tcf4*tg, 6.397 Hz ± 0.7007 n=30, p=0.843; wt, 7.595 Hz ± 1.051 n=41; *Tcf4*Ex4δ^+/-^ 7.813 Hz ± 0.9946 n=39, p=0.768).

l,m) Short-term depression was induced upon high-frequency stimulation at 10 Hz, with no change in the paired-pulse ratio in either of the mutations tested. Paired-pulse ratio: (i) wt, 0.9407 ± 0.02223 n=43, *Tcf4*tg, 0.9692 ± 0.02631 n=43, p=0.540*;* (j) wt, 0.8720 ± 0.02670 n=57, *Tcf4*Ex4δ^+/-^, 0.8996 ± 0.02367 n=51, p=0.183).

Data represent mean±SEM, numbers of measured neurons indicated as inlets of the bar graphs, all pairwise statistical evaluations were performed using the Mann-Whitney test (littermate wt controls vs *Tcf4*tg and littermate wt vs *Tcf4*Ex4δ^+/-^, respectively).


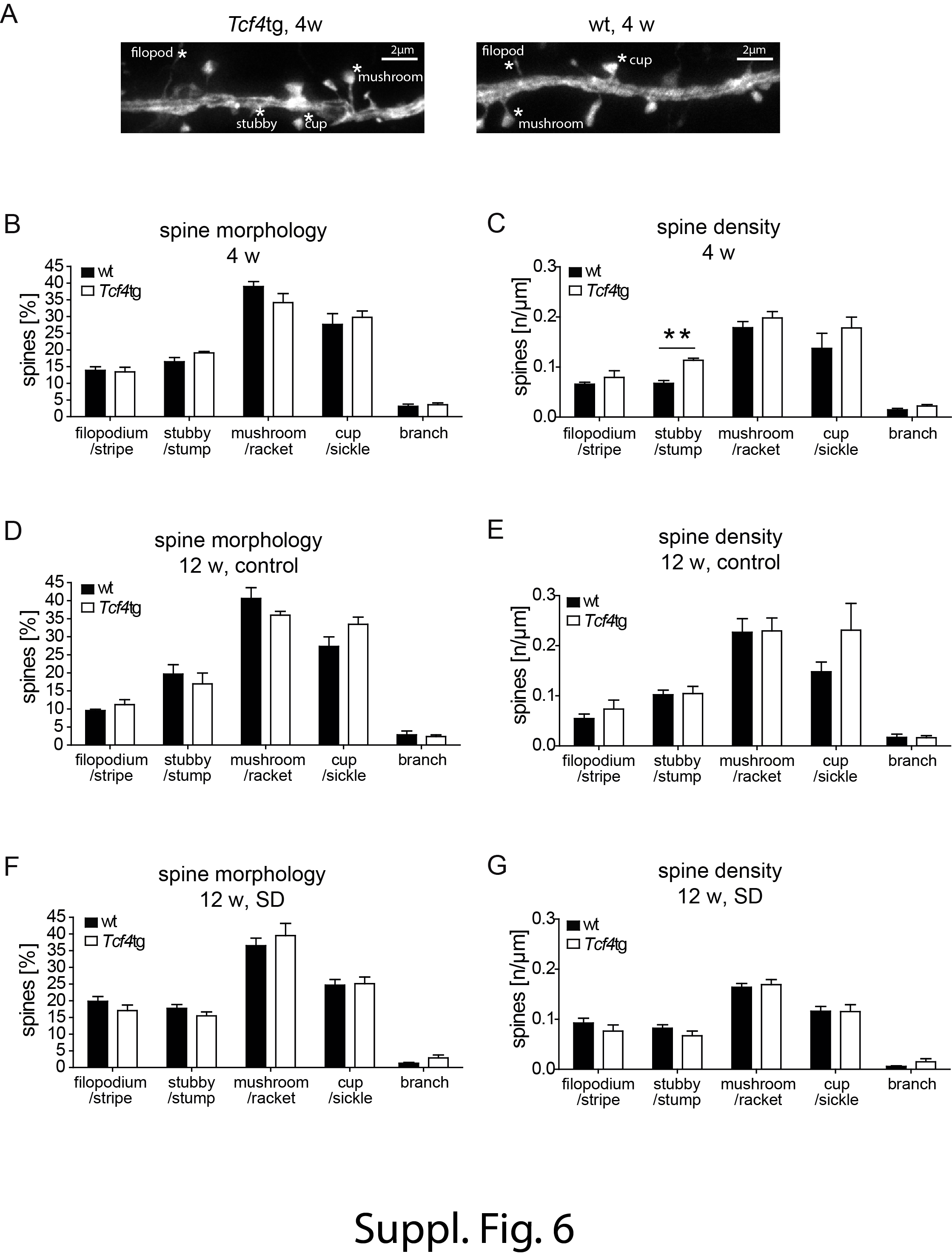


**Suppl. Fig. 6 STED nanoscopy reveals increased immature spine densities in the prefrontal cortex of young adult *Tcf4*tg mice.**

a-c) Analysis of spine structures of 4 weeks old *Tcf4*tg and wt mice.

a) Representative pictures of spine morphology separated into different classes with increasing morphological complexity (filopodium/stripe, stubby/stump, mushroom/racket, cup/sickle, branch) according to [42].

b-c) The relative numbers of spine types were similar between the genotypes (effect of genotype p=0,9997, interaction p= 0.2296)(b) but the density of spines given as absolute numbers per µm of dendrite was increased in *Tcf4*tg mice (effect of genotype p=0.0055; interaction p=0.5657) with the most prominent difference in the density of immature ‘stubby’ spines (p<0.01, post-hoc).

d-e) In 12 weeks old animals from standard housing conditions spine morphology (d) and density (e) was unaltered in both genotypes (morphology: effect of genotype p=0.9997, interaction p=0.0981 and density: effect of genotype p=0.1569, interaction p=0.3466).

f-g) Relative numbers of different morphological spine types (f) and spines density (g) were unaltered in 12 weeks old *Tcf4*tg mice after SD (morphology: effect of genotype p=0.9995 and interaction p=0.4297; density: effect of genotype p=0.5362 and interaction p=0.4975).

Numbers of analyzed mice and dendrites per mouse: b-c) *Tcf4*tg n=7, wt n=5; 19 dendrites each. d-e) *Tcf4*tg, n=4, wt=4; 15 dendrites each. f-g) *Tcf4*tg, n=11, wt, n=7), 12 dendrites each. Data are presented as mean mean±SEM, all group comparisons were performed with ANOVA followed by Bonferroni post-hoc tests.


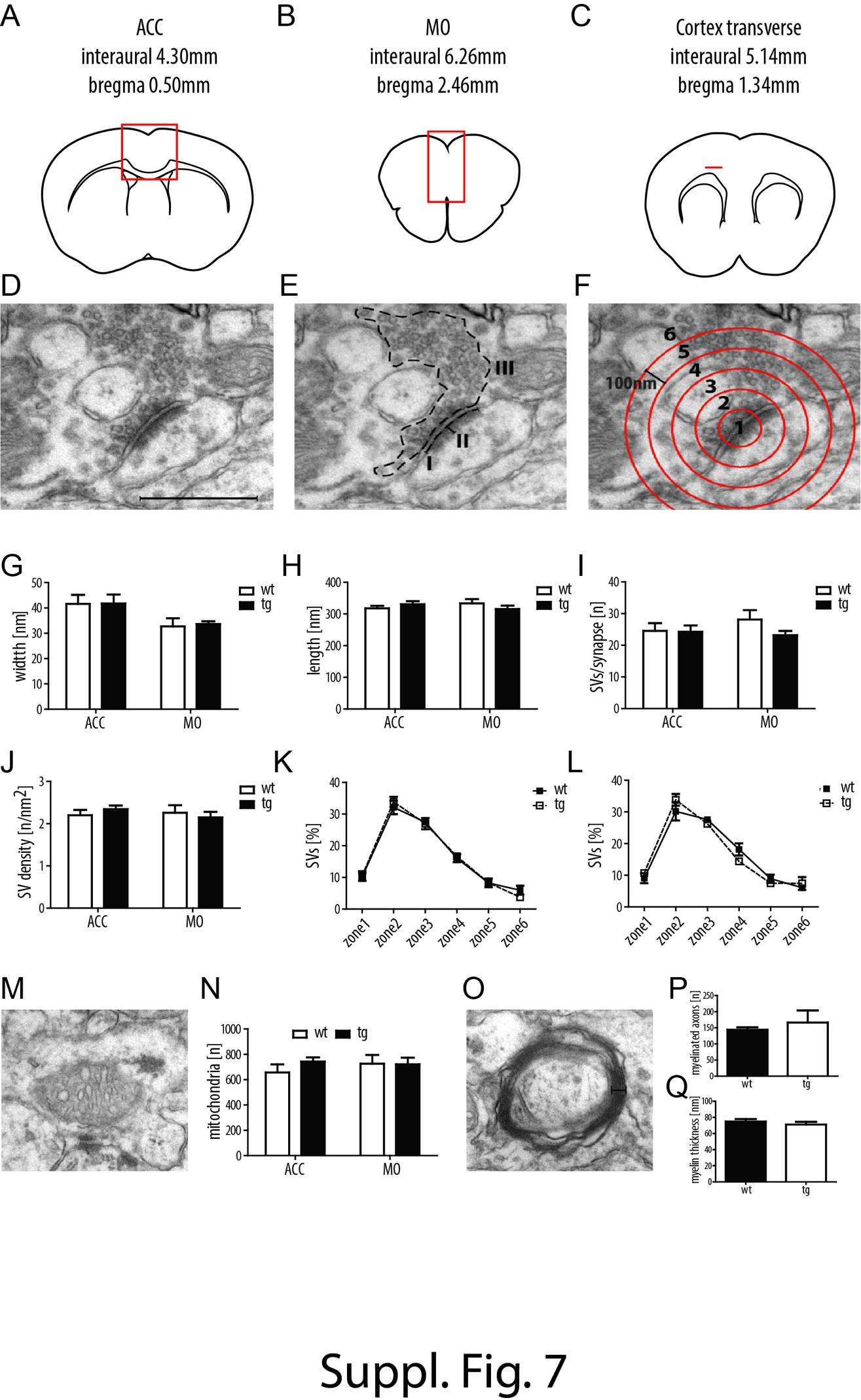


**Suppl. Fig. 7 Electron microscopic analysis reveals no changes in numbers and features of mature synapses, mitochondria and myelin between *Tcf4*tg and wt mice at 4 weeks of age.**

a-c) Electron microscopic analyses of synapse morphology and mitochondria abundance was examined in the anterior cingulate cortex (ACC) (a) and medial orbitofrontal cortex (OFC) (b) and myelin abundance was analyzed in transverse cortical sections (c) in tissue blocks from the indicated coordinates.

(d-e) EM picture of an exemplary asymmetric synapse (size bar = 500 nm) (d) and corresponding analysis strategies (e,f).

g-i) Analysis of the width (g) and length (h) of the synaptic active zone and area occupied by synaptic vesicles (i) following parameters I,II,II depicted in (e), respectively.

j-l) Densities of synaptic vesicles from the active zone were measured by counting numbers of synaptic vesicles in the complete active zone (j) and each of the 6 zones in ACC (k) and OFC (l) as marked in (f).

m) An exemplary mitochondrion.

n) Numbers of mitochondria were counted in ACC and MO. No difference between the genotypes was found.

o) An exemplary myelin sheet. p-q) Numbers of myelinated axons (p) and thickness (q) measured in the cortical transverse region (see c) were unchanged by the genotype.

Number of animals per genotype = 6; 20 sections per genotype and animal were analyzed.

**Suppl. Table 1 The table contains a joint display of all behavioral analyses of TCF4tg and Tcf4Ex4δ+/- mice in comparison to wild-type mice in environments with different stress-levels.**

Statistics depicted are from the multivariate analyses, details are given below the dataset.

**Suppl. Table 2 The table contains hits from the differential mass-spec analysis of synaptosomal and cytoplasmic preparations from FCx of wt and Tcf4tg mice.**

Ration is given as fold-change comparing mean values of all peptides identified. Light red indicates candidate proteins to be expressed at elevated and light blue at reduced levels in Tcf4tg versus wt samples. Darker red and blue indicate candidates found in both subcellular preparations.

**Supplemental Material and Methods**

*Mouse strains*

**C57Bl/6N** wildtype mice, purchased from Charles River (Sulzfeld, Germany), were used for breeding and as strangers in Social Interaction test. **FVB/N**, purchased from Charles River (Sulzfeld, Germany), were used for breeding and as residents in SD paradigm. ***Tcf4*tg** mice kept on FVB/N background were bred to heterozygosity with C57Bl/6N mice and used as F1 C57Bl/6NxFVB/N hybrids for all behavioral experiments, ***Tcf4*tg/bl6** backcrossed for more than 10 generations on C57Bl/6N background were used for STED imaging (see below). **Ella-Cre** line B6.FVB-Tg(EIIa-cre)C5379Lmgd/J was purchased from Jackson Laboratory (stock number: 003724). Cre-recombinase expression starts in all body cells before implantation in the uterine wall. *Tcf4* exon 4 floxed mouse line **(*Tcf4*ex4fl/fl)** MDXP EPD0103 3 A07, (C57Bl/6N background) from **the Sanger** Institute, carrying the EUCOMM allele *Tcf4*^tm1a(EUCOMM)Wtsi^ (project ID: 26368). ***Tcf4*Ex4δ^+/−^** hypomorphic mice were generated by breeding *Tcf4*ex4fl/fl mice to Ella-Cre mice, which caused deletion of exon 4 retaining the lacZ-neo cassette following the knockout-first strategy as described by (Ma et al., 2018)(Figure S 1A). In such obtained offspring, which has C57Bl/6N background, *Tcf4* is heterozygously disrupted in all body cells from early development. **TYFB** mice (on C57Bl/6N background) expressing EYFP under *Thy1.2* promoter (Urban *et al*, 2011) were bred with ***Tcf4*tg/bl6** mice and used for STED imaging.


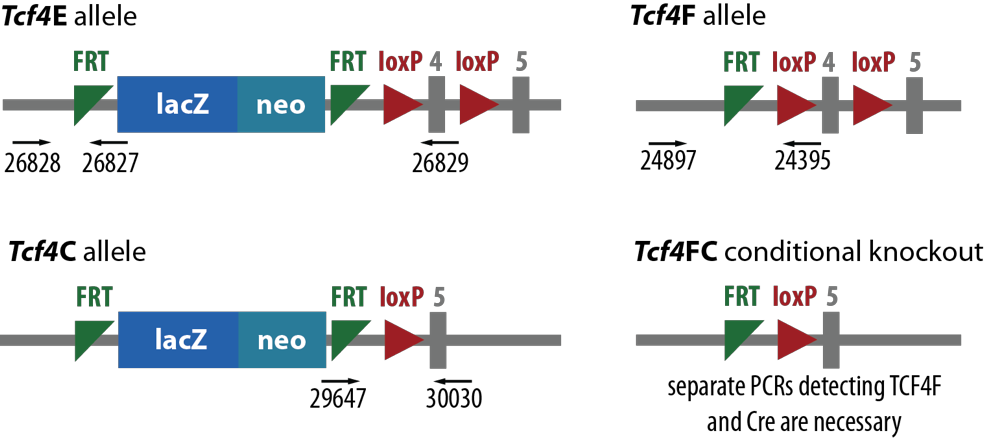
*Genotyping*

Genotyping was performed essentially as described in (Brzózka *et al*, 2010; Brzózka & Rossner, 2013) using the following primers for the mouse strains as indicated below. *Tcf4*ex4fl/fl (‘Tcf4E allele’) and *Tcf4*Ex4δ (‘Tcf4C allele’) lines were genotyped with primer pairs Ex4fl and Ex4δ (see below).

*Primer pairs used:*

EllaCre: F-4192 CAGGGTGTTATAAGCAATCCC, R-4193 CCTGGAAAATGCTTCTGTCCG; product 550bp

*Tcf4*tg: F-4873 TCATAGCCGTCTCAGCAGCCAACCGC, R-4872 CATCGTGTTGCGCAAGAGCCGCGG; 140bp

*Tcf4*Ex4fl/fl: F-26828 CCGATGACAGTGATGATGGT, R-26827 TCGTGGTATCGTTATGCGCC; 172bp

*Tcf4*Ex4δ: F-29647 TCAGCCATATCACATCTGTAGAGG, R-30030 AAATGACTTCCCGCCAGAC; 497bp

*Tcf4*wt: F-26828 CCGATGACAGTGATGATGGT, R-26829 AAGTTAAGCTGAAGTAAATACCCACA; 300bp

TYFB: F-4858 CGCTGAACTTGTGGCCGTTTACG; R-4859 TCTGAGTGGCAAAGGACCTTAGG; 300bp

*Housing conditions*

The temperature in the colony room was maintained at 22±1°C in the 12-hour light-dark cycle. All animals had access to water and food ad libitum. All behavioral experiments were conducted in the light phase (Zeitgeber times [ZT]2 –ZT10), with lights-on at 8:00 AM. Mice were habituated to the experimental room at least 1 week before behavioral experiments. All behavioral experiments were conducted by an investigator blinded to genotype and in accordance with the German Animal Protection Law. For behavioral and experimental analyses, the following housing conditions were applied:

**Isolation rearing (IR)**: From the age of 4 weeks animals were housed individually in Makrolon 2 cages (26.5 x 20.5 x 14.5 cm) that contained only the bedding, noo tissue or other materials that could enrich the cage were provided and animals were handled only during the cage change, as previously described (Badowska *et al*, 2014).

**Enriched environment (EE)**: From the age of 4 weeks animals were group-housed (usually 5–8 mice per cage) in Makrolon 4 cages (60 x 38 x 20 cm) as described (Badowska *et al*, 2014). Cages were divided into two compartments: bigger compartment containing a running wheel and tunnels made of PCV pipe fittings and smaller compartment providing access to food pellets and drinking water. Animals could freely move between the compartments by climbing a ladder or passing through a one-way gate.

**Social defeat (SD)**: To induce psychosocial stress, the resident-intruder paradigm, was used as described (Badowska *et al*, 2014). Single-housed male FVB/N mice (Charles River, Sulzfeld, Germany) were used as residents. In brief, from the age of 4–5 weeks the experimental animals (intruders) were introduced in the cages of residents. After the first attack, each intruder was protected by a wire mesh cage to prevent injuries and left in the resident’s cage for 1 h. The procedure was repeated daily for 3 weeks and every day intruder mice were exposed to different residents in a Latin-square manner. Between and after the stress sessions the intruders were housed individually (cages contained bedding and tissue) to prevent abolishment of stress effects by social support. The FVB/N residents were kept in a separate room to avoid olfactory habituation in experimental mice.

**Handling Control (HC)**: mice were housed individually in the same conditions as the SD group, but were handled daily for 2-3 min. HC group served as control to SD procedure.

*Behavioral cohorts*

**Cohort 1**: *Tcf4*tg IR-EE: 59 male *Tcf4*tg and wt mice were housed in IR or EE from the age of 4 weeks and tested from the age of 8 weeks. The cohort consisted of 16 wt IR, 15 *Tcf4*tg IR, 16 wt EE and 12 *Tcf4*tg EE animals. Order of tests: LD, OF, HB, Y-maze, PPI, Social Interaction, TST, FC, HP, MWM. **Cohort 2**: *Tcf4*tg SD-HC: 59 male *Tcf4*tg and wt mice were subjected to SD or HC from the age of 3 weeks and tested from the age of 7 weeks. The cohort consisted of 15 wt SD, 15 *Tcf4*tg SD, 15 wt ctrl and 14 *Tcf4*tg ctrl animals. Order of tests: Social avoidance, Social interaction, EPM, LD, OF, HB, TST, Y-maze, FC, MWM. **Cohort 3**: *Tcf4*Ex4δ*^+/-^* IR: 30 male mice (14 wt, 16 *Tcf4*Ex4δ*^+/-^*) were housed in IR from 4 weeks of age and tested from the age of 10-13 weeks. Order of tests: LD, EPM, OF, HB, Y-maze, Social Interaction, TST, FC, WM (variant with DMP), PPI, remote FC, HP.

See Fig. 1a for illustration and below for behavioral tests. **Abbreviations:** Fear conditioning (FC), Morris water maze (MWM), Open field (OF), Light-dark preference (LD), Elevated plus maze (EPM), Hole board (HB), Tail suspension test (TST), Hot plate (HP), PPI (Pre-Pulse Inhibition).

*Behavioral profiling*

The overall approach was described previously (Badowska *et al*, 2014). Analyses were done in R software version 2.15.2 using the R-package *nlme* and R-functions *gls* and *anova*. Graphs were generated using R-package *plotrix*, exported as .eps files and edited in Adobe Illustrator CS5.

The data calibration and dimensional reduction strategy involved the following steps:

1. **Directionality:** Different behavioral parameters, called measures, are expressed in different units (e.g. seconds, meters, indexes etc.). To allow comparisons, raw data were given a common directionality (i.e. multiplied by 1 or -1; column ‘Directionality’ in Table S 1) such that all negative deviations from the ‘norm’ (wild type in control environment) can be regarded as impairments.
2. **Data calibration:** Experimental groups were calibrated to appropriate controls (e.g. to wt/EE) using z-transformation. This scaled the control group to zero mean and variance one and the data of experimental groups relative to the controls.
3. **Reduction to traits:** Measures comprising similar behavioral phenotypes were merged into single sum scores called traits, e.g. exploration time and nose pokes in HB were compressed to HB-exploration.
4. **Reduction to domains, superdomains and symptom classes:** Traits reflecting similar behaviors were analyzed together as single domains by using multivariate statistics, e.g. OF-time in the center, Light-Dark preference and EPM-anxiety were analyzed collectively as Anxiety. Next, the reductions were stepwise increased into superdomains and symptom classes (Table S 1 and below), which could potentially be compared to clinical symptom classes of psychiatric diseases.
5. **Visualization of behavioral profiles:** Estimated effect sizes in calibrated data from the different levels of hierarchal data reductions were visualized by radar charts as indicated.

*Multidimensional statistics*

Statistical comparisons by ANOVA were done in a hierarchical order - first at the domain level and then, if significant, at lower levels. This approach interpreted data towards clinical symptom domains, increased statistical power and accounted for multiple testing. In detail: Raw data were given a common directionality (multiplied by 1 or -1; Table S 1, column ‘Directionality’) and within-experiment data standardization by z-transformation was used with internal reference: wt-EE (cohort 1: 4-arm experiment IR/EE × tg/wt), wt-HC (cohort 2: 4-arm experiment SD/HC × tg/wt), wt^†^-IR (cohort 3: 2-arm experiment IR × ko/wt^†^). Tested were three symptom classes (*cognition*, *negative symptoms*, *positive symptoms*; Bonferroni p≤0.05/3=0.0167) that divide into eight superdomains (*spatial learning*, *fear memory*, *working memory; pain sensitivity*, *anxiety*, *curiosity*, *motivation*; *hyperactivity;* Bonferroni p≤0.05/8=0.00625) and further divide into 14 domains (Bonferroni p≤0.05/14=0.00357); see Table S 1. Significance was determined by using the Bonferroni threshold combined with the closed testing principle. Hence the global test (ANOVA: all data) was performed prior to experiment-wise and pairwise tests within experimental units (cohorts); tests of symptom classes prior to tests of superdomains, followed by tests of domains, and finally by tests of single measures. At each data compression step, a multivariate test (joint test of all relevant measures) as well as a univariate test of the sum score (i.e., the individual mean of all relevant measures) were performed. Both tests are displayed (Table S 1). For the establishment of significance, it was sufficient that at least one of these two highly correlated statistical tests passed the respective significance threshold. In the testing of domains (or: superdomains, symptom classes), multivariate traits (or: domains, superdomains) were represented by the univariate sum score of relevant measures (or: traits, domains), respectively. Multivariate tests are more powerful, e.g., when all measures (or: traits, domains, superdomains) that contribute to a trait (or: domain, superdomain, symptom class) are consistently affected by environmental or genetic factors. Univariate (sum score) tests can be more powerful when only some measures (or: traits, domains, superdomains) are affected. Effects with different directionality (e.g., SD effects on superdomains of the negative symptom class) diminish significance in the next higher data compression level.

*Behavioral tests*

Behavioral tests were in general following published procedures (Badowska *et al*, 2014; Brzózka *et al*, 2010). The experimental chambers and mazes were washed with 70% ethanol before and after each use, unless stated differently.

**Open field (OF)** and **Hole board (HB)**: Animals were placed into a grey box (45 x 45 x 55 cm) and allowed to explore the surrounding for 10 min. In the OF test, time moving, covered distance, rearing and time in the centre were quantified using an infrared monitoring system and the Moti4 software (TSE Systems, Bad Homburg, Germany). The HB experiment was performed in the same boxes, but with a floor insert containing 16 symmetrically deployed holes (2 cm diameter). During the 10 min testing phase, the number of nose pokes into the holes and total time of hole exploration were measured automatically by the Moti4 software.

**Light-dark preference (LD)**: The experiment was performed in a chamber divided into two compartments: black-walled “dark” chamber and transparent “light” chamber, both connected by a door-like opening. Mice were placed into the light chamber, with their heads facing the wall opposing the gate. The test lasted 5 min from the first entry into the dark chamber. The latency to enter the dark chamber and the total time spent there were measured manually.

**Elevated plus maze (EPM)**: The EPM setup was built in a shape of a “plus” sign with two opposing open and two closed arms (305 cm arms, walls 15 cm high) and raised 50 cm above the floor. Each animal was placed at the crossing of the arms. The time spent in the open and closed arms were manually measured for 5 min.

**Tail suspension test (TST)**: Mice were suspended upside-down and attached to a fixed rod by an adhesive tape by the tip of the tail. Fighting time, which reflects the escape motivation of the mice, was manually scored for each mouse for 6 min.

**Y-maze**: Mice were inserted into a gray plastic maze in the shape of “Y” with arms identical and symmetric to each other. Animals were allowed to explore the maze for 10 min. The number of arm explorations (choices) and number of alternations were scored. Alternation was defined as a sequence of thee arms explorations without visiting the same arms twice.

**Social interaction**: We used the Crawley test of sociability to analyze social behavior (Moy *et al*, 2004). The test box consisted of three compartments separated by transparent plexi walls with entrances. In the acquisition phase (5 min) the experimental animal was placed in the middle, empty compartment and the entrances to other compartments were blocked. Next, in the sociability phase, an unfamiliar mouse (stranger 1) was introduced to one of the compartments and covered by a wire-mesh cage. And empty wire mesh cage was placed in the opposite compartment. The experimental mouse was allowed to explore all compartments for 10 min. In the last phase (social memory) another unfamiliar mouse (stranger 2) was placed in the previously empty wire-mesh cage and the experimental animal was allowed to explore the box for 10 min. Experiments were recorded by a camera placed above the test box and the time spent in each of the side compartments was then manually measured. Sociability and memory indexes were calculated according to the formulas: sociability index = (t_s_1/ t_s_1+ t_e_)+50; memory index = (t_s_2/ t_s_1+ t_s_2)+50; where t_s_1 and t_s_2 are times spent in the compartments with stranger 1 and stranger 2 and t_e_ is the time in the compartment with the empty wire-mesh cage. All stranger mice were C57Bl/6N males younger than the experimental mice. To avoid any repulsive stress or anxiety signals from the strangers, before the experiment they were habituated to the wire-mesh cages several times and during the experiment different pairs of strangers were used in consecutive sessions, to let the mice recover.

**Fear conditioning (FC)**: The test was performed as described in described (Brzózka *et al*, 2010; Brzózka & Rossner, 2013). Commercial fear conditioning systems used: TSE Systems (Bad Homburg, Germany) for cohorts 1 and 2 and Ugo Basile (Siena, Italy) for the cohort 3. The procedure was performed as with the TSE System with following modifications: i) freezing was recorded by an infra-red CCD camera (47400-025) and measured automatically by the Any-maze software (cat.no. 60000-FC); ii) shocks were applied in the Ugo Basile 46003 Mouse Boxes (inside dimensions: 17 x17 x 25(h) cm with the vertical stripe patterns on the walls; iii) for cue memory Mouse Boxes were replaced by a transparent Plexiglas cylinders (diameter 19.5 cm, height 25 cm).

**Morris water maze (MWM)**: The test was performed as described (Brzózka *et al*, 2010) in a white pool (diameter 120 cm) filled with water dyed with white paint. A white platform (diameter 10 cm) was located in one of the target quadrants, 1 cm under the water surface. To allow navigation, a single cue was placed on the wall. The animals’ position, time, distance, route and speed of swimming were tracked using TSE VideoMot-Systems. The test consisted of several phases and different behavioral qualities were tested in each phase. Learning curves. For several consecutive days’ mice had four swimming trials per day, each time at the different pole of the pool (the order of the poles was different every day). Animals were tested in batches of 4–5 mice, so the trials were separated by intervals of around 5 min. During each trial (max. 90 s long) animals were supposed to find for the hidden platform and remain on it for 10 s. If a mouse failed to find the platform, it was gently guided to it. The means of all four trials were used to draw the learning curves. The test was composed of an initial acquisition phase - visible platform (2 days) -, an initial learning - hidden platform (6 days) - where the animals had to navigate based on the position of the cue, showing spatial learning abilities, and a reversal learning phase (6 days) where the platform was moved to the opposite quadrant to assess flexibility learning. Memory recall was assessed in probe tests (1 day) in which the platform was removed and mice were allowed to swim in the pool for 90 s in a single trial. The time and distance spent in the target quadrant were recorded. Probe tests were performed after initial and reversal (flexibility) learning and a remote probe test 1 month later.

**Hot plate (HP)**: Thermal pain sensitivity was assessed by putting mice on a hot plate preheated to 52°C. The latency until licking the hind paws or jumping was measured. Afterwards animals were immediately removed from the hot plate and put on a metal top to cool down their paws. Pain threshold Pain sensitivity to electric shocks was measured in the TSE System that was used for Fear conditioning. Animals were placed on the shock grid and a series of 2 s electric shocks of different intensities (0.1–0.7 mA) was applied. The shock intensities were presented in a randomized order and with randomized intervals between them. The animals were observed and the lowest shock intensities that induced reaction (jumping, vocalizing) were noted.

*Electrophysiology*

**Autaptic cultured neurons:** Microisland autaptic culture preparation was performed according to published procedures (Burgalossi *et al*, 2012). Cells were whole-cell voltage clamped at -70 mV with an EPC9 (HEKA) amplifier under the control of the PatchMaster program. Data analysis was performed using Axograph X. The cells were recorded between DIV10-14. EPSCs were evoked by a 2-milisecond depolarizing pulse from -70 mV to 0 mV. The response to triggered release of the RRP was measured after application of hypertonic sucrose solution (500 mM). mEPSCs were recorded in the presence of 300 nM TTX. The extracellular solution contained (in mM): 140 NaCl, 2.4 KCl, 10 HEPES, 10 glucose, 4 CaCl_2_ and 4 MgCl_2_ (320 mOsmol/l, pH 7.3). The patch-pipette solution for autaptic recordings contained (in mM): 136 KCl, 17.8 HEPES, 1 EGTA, 4.6 MgCl_2_, 4 NaATP, 0.3 Na_2_GTP, 15 creatine phosphate and 5 U/ml phosphocreatine kinase (315-320 mOsmol/l, pH 7.4). The series resistance was compensated by between 60-80%. All extracellular solutions were applied with a custom-built fast flow system consisting of an array of flow pipes controlled by a stepper motor that allows complete and rapid solution exchange with time constants of approximately 30 ms. For statistical analysis a normality test was run followed by either T-test or Mann-Whitney U test correspondingly.

**Acute brain slices:** LTP and LTD were measured in transverse hippocampal slices from 4-week-old mice. Schaffer collateral afferents were stimulated and field excitatory postsynaptic potentials (fEPSPs) were measured in the stratum radiatum of CA1 with a GABA inhibitor. LTP was induced by 1 s of high frequency stimulation and LTD by applying low frequency stimulation for 15 min. fEPSP slopes were expressed relative to normalized baseline. For Input-output curves mean fEPSPs from three consecutive responses were used. Mice were anesthetized with Isofluorane (DeltaSelect) and decapitated. Brains were removed from the skull and placed into a sucrose-based slicing solution on ice (230 mM Sucrose, 26 mM NaHCO_3_, 2 mM KCl, 1 mM KH_2_PO_4_, 1 mM MgCl_2_, 10 mM Glucose, 0.5 mM CaCl_2_) and oxygenated with carbogen gas. Transverse hippocampal slices (300 μm thick) were obtained using a Mcilwain tissue chopper (U0800; Lafayette instrument). The sections were transferred to a holding chamber and maintained in ACSF (120 mM NaCl, 26 mM NaHCO_3_, 1 mM KH_2_PO_4_, 2 mM KCl, 2 mM MgCl_2_, 10 mM Glucose, 2 mM CaCl_2_). Before recording, sections were allowed to recover for 60 min. Evoked field excitatory postsynaptic potentials (fEPSPs) were obtained using a glass-recording electrode filled with ASCF and placed the stratum radiatum (CA1 recordings). fEPSP were collected using an Axon MultiClamp 700B amplifier and pCLAMP software (Molecular Devices). Slopes from individual fEPSP traces were calculate from the initial slope of the fEPSP relative to the slope of the 10ms interval immediately preceding afferent stimulation. Data were analyzed using Axograph X software (Axograph Scientific) and ANOVA was applied for statistical analysis.

*Super-resolution light microscopy via STED nanoscopy*

The stimulated emission depletion (STED) nanoscopy experiment was performed essentially as described (Agarwal *et al*, 2014) with minor modifications. To asses spine numbers and morphology, *Tcf4*tg mice were bred to TYFB mice that express EYFP in postnatal forebrain under Thy1.2 promoter as described above. Double tg mice were anesthetized by intraperitoneal injections of pentobarbital (120 mg per kg body weight) and Buprenorphine (2 g) and perfused with 4% PFA in 0.1M phosphate buffer (pH 7.4). After an overnight postfixation in 4% PFA at 4°C, the brains were cut on a Vibratome (VT 1000S, Leica) into 70 µm sections. Images of spine morphology were taken in ACC using a home-built STED microscope (Urban *et al*, 2011). Recording of image stacks x-y-z (18 µm x 18 µm x 3 µm) was performed with a STED resolution around 60 nm and pixel dwell time of 10 µs. Images of dendrites parallel to the slice surface were processed using the “Simple Neurite Tracer” function of ImageJ or Fiji software. *Tcf4*tg x TYFB male mice were analyzed at the age of 4 weeks and 12 weeks housed under control condition or subjected to social defeat. Statistical analyses were done with Mann-Whitney U test for pairwise comparisons and ANOVA for subtype analysis.

*Proteomics on cytosolic fractions and synaptosomes*

Synaptosomes were isolated according to (Gray & Whittaker, 1962; Biesemann *et al*, 2014). All centrifugation steps were done at 4°C and samples were kept on ice between steps. 4 weeks old male *Tcf4*tg (n=4) and wt mice (n=4) were sacrificed and prefrontal cortex (600–900 mg) was isolated, washed with ice-cold PBS and homogenized 10 times by 12–15 up and down strokes in 900 l of ice-cold buffer A supplemented with phosphatase inhibitor cocktails I and II (Sigma, 1 l per 100 l buffer). Left PFC was pooled for genotypes in 900 l buffer A and right PFC was treated individually for each animal. Homogenate was centrifuged at 3200 rpm for 10 min. Supernatant (S1-nuclei) was transferred to a different tube using a 200µl pipette with a cut tip and kept on ice. Pellet (P1-cell debris) was resuspended in 800 l of buffer A (without phosphatase inhibitors) and centrifuged at 3200 rpm for 10 min. Supernatant S1’ was combined with S1 and 100 l S1 was saved for further analysis. Pellet (P1) was resuspended in 800 l buffer A and 100 l was saved for further analysis. The supernatant S1 was centrifuged at 11500 rpm for 15 min and 100 l of supernatant (S2) was saved. Pellet (P2) was carefully resuspended with a pipette in 1 ml of homogenization buffer (0.32M sucrose) and pipetted on top of a discontinuous sucrose density gradient (from bottom: 4 ml 1.2 M, 4 ml 1M and 3 ml 0.8 M) and centrifuged in and Ultracentrifuge with rotor Sv40Ti 2 h at 25000 rpm. The synaptosomal fraction, obtained from 1.2–1M interphase, diluted 1:1 in water (water added drop by drop with mixing) and centrifuged 20 min at 30000 rpm in polyallometer centrifuging tubes. The pellet (S4, synaptosomes) was resuspended in 10 l water for proteomic or in 50 l for western blotting and stored at -20°C. Proteome analysis of cytosolic fractions (S1) and synaptosomes (S4) and the western blots were performed according to established protocols (Maccarrone *et al*, 2010; Agarwal *et al*, 2014). In brief: samples (100 g total protein) underwent isotope-coded protein labeling (ICPL)195 and 50 g proteins were prefractionated by on a 12% SDS-PAGE minigel. Shotgun mass spectrometry was performed, and proteins were identified using an in-house version of MASCOT Distiller 2.2.3 software (Matrix Sciences, London, UK) and searched against a decoy Uniprot mouse protein database (release 2012 06). Proteins were considered as differentially expressed when they had more than 2-fold change or 1.5 when quantified by minimum 5 peptides. Then, proteins were divided to classes based on Human Protein Reference Database [http://www.hprd.org](http://www.hprd.org/). Hits (Table S 2,3) were analyzed for protein interactions using the Bisogenet plugin from Cytoscape Apps which consolidates data from DIP, BIOGRID, HPRD, BIND, MINT and INTACT databases. The resulting network was further filtered to nodes with direct interaction to hit-list nodes. Edges between the non-hit-list nodes were removed to reduce the complexity of the network. Node attributes and pathway annotations were added based on KEGG pathways as described (Herholt *et al*, 2018).

*Electron microscopy*

Four-week-old mice *Tcf4*tg (n=5) and wt littermates (n=5) were anaesthetized and perfused with 15 ml of Hanks balanced salt solution (HBSS, PAA laboratories, Pasching, Austria) and then by fixative as described in197 using a Heidolph PD5201 Peristaltic Pump. The brain tissue was dissected and 200 µm coronal sections were cut with a Leica VT1200S Vibratom (Leica Microsystems,Wetzlar). The medial orbitofrontal cortex (MO), anterior cingulate cortex (ACC) and cortex transversal areas (Figure S 7A-C) were punched out of the section by using a 2mm Harris Uni-core Punch. After postfixation with 2% OsO4 (Science Services, Munich, Germany) and dehydration with ethanol and propylenoxid (automated system EMTP Leica Microsystems, Wetzlar) samples were embedded in Epon (Serva) and cut in the microtome (Ultracut S, Leica). Semi-thin (500 nm) and ultra-thin (50 nm) sections were prepared using diamond knifes (Histo 45and Ultra 45, Diatome Biel CH). Semi-thin sections were collected onto a glass slide and dried on a 60 C hot plate to verify the area of interest by using a Leica Dialux 20 light-microscope. Ultra-thin sections were placed on 100 mesh hexagonal copper Grids (Gilder Grids Ltd. Grantham UK) coated with “Formvar” (Plano Wetzlar) and stained with Uranylacetat (SPI-Chem West Chester,USA) and Lead citrate (Merck, Darmstadt)(REYNOLDS, 1963). Ultra-thin sections were analyzed using a Zeiss EM900 Elektron-Microscop (Zeiss, Oberkochen, Germany) with the 3000, 12000 and 30000 magnification. Digital pictures were taken by the wide-angle dual speed 2K-CCD-Camera (TRS, Moorenweis, Germany). Pictures of transverse MO and ACC regions were taken at 12000 and 30000 magnifications. Total number of synapses, perforated synapses and mitochondria was counted within 20 randomly taken images under 12000 magnification. The synapse structure was analyzed under 30000 magnification with 50 synapses per animal. The average length and width of the active zones, number of synaptic vesicles per synapse and the synaptic vesicle cluster density (number of vesicles divided by the area they occupy) were calculated separately for symmetric and asymmetric synapses. Additionally, the average distance of the vesicles from the active zone was measured using the Concentric Circles plugin for ImageJ software: 5 differently sized circles with the center in the middle of the active zone were overlaid on the synapse images. Circles divided the synapse area into 5 zones, each 100 nm wide. Zone 6 is the area outside of the biggest circle, more than 500 nm away from the active zone. The number of vesicles in each zone was counted for each synapse and the averages were calculated. For statistical analysis a normality test was run followed by either t-test or Mann-Whitney U test correspondingly.

**Supplemental References**

Agarwal A, Zhang M, Trembak-Duff I, Unterbarnscheidt T, Radyushkin K, Dibaj P, Martins de Souza D, Boretius S, Brzózka MM, Steffens H, Berning S, Teng Z, Gummert MN, Tantra M, Guest PC, Willig KI, Frahm J, Hell SW, Bahn S, Rossner MJ, et al (2014) Dysregulated Expression of Neuregulin-1 by Cortical Pyramidal Neurons Disrupts Synaptic Plasticity. *Cell Rep.*

Badowska DM, Brzózka MM, Chowdhury A, Malzahn D & Rossner MJ (2014) Data calibration and reduction allows to visualize behavioural profiles of psychosocial influences in mice towards clinical domains. *Eur. Arch. Psychiatry Clin. Neurosci.***:** 1–14

Biesemann C, Grønborg M, Luquet E, Wichert SP, Bernard V, Bungers SR, Cooper B, Varoqueaux F, Li L, Byrne JA, Urlaub H, Jahn O, Brose N & Herzog E (2014) Proteomic screening of glutamatergic mouse brain synaptosomes isolated by fluorescence activated sorting. *EMBO J.* **33:** 157–170

Brzózka MM, Radyushkin K, Wichert SP, Ehrenreich H & Rossner MJ (2010) Cognitive and sensorimotor gating impairments in transgenic mice overexpressing the schizophrenia susceptibility gene Tcf4 in the brain. *Biol. Psychiatry* **68:** 33–40

Brzózka MM & Rossner MJ (2013) Deficits in trace fear memory in a mouse model of the schizophrenia risk gene TCF4. *Behav. Brain Res.* **237:** 348–356

Burgalossi A, Jung S, Man KM, Nair R, Jockusch WJ, Wojcik SM, Brose N & Rhee J-S (2012) Analysis of neurotransmitter release mechanisms by photolysis of caged Ca^2+^ in an autaptic neuron culture system. *Nat. Protoc.* **7:** 1351–1365

Gray EG & Whittaker VP (1962) The isolation of nerve endings from brain: an electron-microscopic study of cell fragments derived by homogenization and centrifugation. *J. Anat.* **96:** 79–88

Herholt A, Brankatschk B, Kannaiyan N, Papiol S, Wichert SP, Wehr MC & Rossner MJ (2018) Pathway sensor-based functional genomics screening identifies modulators of neuronal activity. *Sci. Rep.* **8:** 17597

Maccarrone G, Turck CW & Martins-de-Souza D (2010) Shotgun mass spectrometry workflow combining IEF and LC-MALDI-TOF/TOF. *Protein J.* **29:** 99–102

Moy SS, Nadler JJ, Perez A, Barbaro RP, Johns JM, Magnuson TR, Piven J & Crawley JN (2004) Sociability and preference for social novelty in five inbred strains: an approach to assess autistic-like behavior in mice. *Genes Brain Behav.* **3:** 287–302

Skarnes WC, Rosen B, West AP, Koutsourakis M, Bushell W, Iyer V, Mujica AO, Thomas M, Harrow J, Cox T, Jackson D, Severin J, Biggs P, Fu J, Nefedov M, de Jong PJ, Stewart AF & Bradley A (2011) A conditional knockout resource for the genome-wide study of mouse gene function. *Nature* **474:** 337–342

Urban NT, Willig KI, Hell SW & Nägerl UV (2011) STED nanoscopy of actin dynamics in synapses deep inside living brain slices. *Biophys. J.* **101:** 1277–1284
